# Closeness to God, Spiritual Struggles, and Wellbeing in the First Year of College

**DOI:** 10.3389/fpsyg.2022.742265

**Published:** 2022-03-31

**Authors:** Madison Kawakami Gilbertson, Shannon T. Brady, Tsotso Ablorh, Christine Logel, Sarah A. Schnitker

**Affiliations:** ^1^School of Psychology, Fuller Theological Seminary, Pasadena, CA, United States; ^2^Department of Psychology, Wake Forest University, Winston-Salem, NC, United States; ^3^Department of Clinical Psychology, University of Massachusetts Boston, Boston, MA, United States; ^4^Department of Social Development Studies, Renison University College, University of Waterloo, Waterloo, ON, Canada; ^5^Department of Psychology and Neuroscience, Baylor University, Waco, TX, United States

**Keywords:** closeness to God, spiritual struggles, spirituality, wellbeing, belonging, college

## Abstract

Spirituality is an important, but oft-overlooked, aspect of the self that may affect college students’ wellbeing and belonging. Few studies have systematically examined closeness to God and spiritual struggles as predictors of college student wellbeing during early college, which is a critical window for identity development. Moreover, research exploring interactions between spiritual struggles and closeness to God in predicting wellbeing outcomes is scarce. We address these gaps in the literature with an analytic sample comprised of 839 first-year college participants who identify as religious. The results of correlational analyses and linear mixed effect models are presented. Closeness to God was associated with greater wellbeing and belonging, and spiritual struggles were associated with lower wellbeing and belonging. In exploratory analyses, a moderating effect of closeness to God on the relation between spiritual struggles and negative outcomes was observed. Implications for higher education and college student development are discussed.

## Introduction

In college, students must find their place within a new physical, social, and academic context. A growing body of research finds that students’ social identities (Tajfel and Turner, 1979), and psychological factors associated with those identities, can consequentially shape students’ wellbeing and belonging early in college and beyond (Walton and Cohen, 2007, 2011; Strayhorn, 2012; Brady et al., 2020). Most research on how social identities influence students’ outcomes during college has focused on racial/ethnic background, gender, or first-generation college status (Walton and Cohen, 2011; Stephens et al., 2012; Walton et al., 2015; Yeager et al., 2016). Other aspects of students’ identities merit further inquiry. Here, we focus on spiritual identity, specifically students’ sense of their closeness to God and the extent to which they struggle spiritually. We examine how these spiritual factors are associated with other important early college outcomes: wellbeing and belonging.

### Religion and Spirituality in College

Even though spiritual identity is of central importance to human development (Barry and Abo-Zena, 2014), it is often ignored by researchers (Astin, 2004). This oversight is glaring, as approximately 71% of United States college students identify as part of a religion (Eagan et al., 2015). Although the level of students’ religious *activity*—such as attending church services—often declines during college (Astin et al., 2011a,b; Barry and Abo-Zena, 2014), the importance of their religious and spiritual *beliefs* remain stable (Hunsberger, 1978; Bryant et al., 2003; Koenig et al., 2008). Moreover, young adults may become more open to spiritual searching and religious diversity (Small and Bowman, 2012). Arnett (2014) noted that emerging young adults seek meaning as they develop their identity, and many look to religious/spiritual sources to construct a meaning system.

As with other social identities, spiritual identity can affect people’s everyday experiences. For example, researchers have found that spiritual factors may not only act as psychological buffers for young adults in the face of stress (Bryant et al., 2003; Yonker et al., 2012) but may also serve as sources of distress that could negatively affect their college outcomes (Exline and Rose, 2005; Exline, 2013; Wilt et al., 2017). In non-college samples, spiritual factors have been linked to important outcomes, like wellbeing (Koenig et al., 2012; Barry and Abo-Zena, 2014).

Most existing research has focused on spiritual identity development during adolescence and emerging adulthood (MacDonald, 2011; Roehlkepartain et al., 2011) or the effects of religious affiliation on wellbeing (Schwab and Petersen, 1990; Tix and Frazier, 2005; Green and Elliott, 2010; Mochon et al., 2011; Small and Bowman, 2012). However, more studies are needed that examine specific religious and spiritual factors related to identity that are of particular importance for determining the negative or positive effects of religion and spirituality on wellbeing (Hardy et al., 2019). This is the approach we adopt in the present study.

Two variables in the literature related to spiritual identity that, to our knowledge, have not been studied together in college populations are *closeness to God* and *spiritual struggles*. Especially early in college, religion and spirituality may serve as a helpful coping resource that alleviates stress in the new collegiate environment through a sense of closeness to God (e.g., a close relationship with God may buffer against stress about making new friends in college; Laurin et al., 2014; Jeppsen et al., 2015), or as a source of stress related to spiritual struggles (e.g., a first-year student has her beliefs challenged and struggles with doubt; Bryant and Astin, 2008).

#### Closeness to God

Hill and Pargament (2003) define closeness to God as an individual’s “felt close relationship with the divine” (p. 67). In many religious traditions, developing a close relationship with God is a key aspect of spiritual development (Kass et al., 1991; Hill and Pargament, 2003), and models of relational spirituality maintain that the quality of personal relationships with the divine is a central factor of many predominant monotheistic religions (e.g., Davis et al., 2018; Verhagen and Schreurs, 2018).

The literature on closeness to God draws from theory on religious coping and attachment. People who report a secure attachment to God—that is, they have a close relationship with God as an attachment figure and view God as a secure base—are more likely to report better health (Krause, 1998) and better psychosocial adjustment. This is especially true in the face of major stressors like health issues (Koenig et al., 1998; Tix and Frazier, 1998) or crisis situations (Smith et al., 2000). Early findings have been replicated in the past two decades; a recent meta-analysis of 123 studies found that a secure attachment to God was positively associated with a variety of outcomes related to wellbeing, self-concept, and positive relationships among theistic participants (Stulp et al., 2019).

The transition to college is a major stressor for many students—one that can last their entire first year of college or longer (Misra and McKean, 2000; Conley et al., 2014, 2020). Drawing on the existing literature, we expect that students’ closeness to God may affect how students respond to stressors like a tense roommate relationship, an overwhelming workload, or challenges to their faith, and thus will be associated with their wellbeing at the end of their first year of college.

#### Spiritual Struggles

Although spiritual struggles can be defined in different ways, we use the definition proposed by Exline et al. (2014) who define it as negative thoughts or conflict associated with some aspect of spirituality (e.g., beliefs, practices, experiences). Spiritual struggles can involve struggles with supernatural agents (deities or demons), struggles with other people or with institutions over religious or spiritual matters, or struggles within oneself regarding morality, doubt, or ultimate meaning in life.

In most studies, spiritual struggles are negatively associated with wellbeing and positively associated with psychological distress, including anxiety, depression, negative outlooks on life, suicidality, and negative outcomes in stressful situations (Pargament et al., 1998; Exline et al., 2000, 2014; Ano and Vasconcelles, 2005; Exline and Rose, 2005; McConnell et al., 2006; Bryant and Astin, 2008; Exline, 2013; Wilt et al., 2017). In a meta-analysis of 32 studies examining the links between spiritual struggles and psychological adjustment across time (Bockrath et al., 2021), a small significant meta-analytic effect for negative psychological adjustment (e.g., depression symptoms, anxiety symptoms, PTSD symptoms) suggests spiritual struggles predict exacerbation of mental illness symptoms and other indicators of poor adjustment. In contrast, there was no significant meta-analytic effect for indicators of positive psychological adjustment (e.g., life satisfaction, optimism, self-esteem).

Spiritual struggles are common in college as students re-examine their religious and spiritual beliefs outside of their familial and community contexts (Barry and Abo-Zena, 2014). For example, students may have trouble integrating new knowledge from their academic courses with their faith tradition. Students of different faith traditions may clash on campus. Or, students may find themselves at odds with other believers within their faith tradition if their spiritual struggles put them at odds with other believers or if the faith community environment discourages discussion of existential questions, ultimate meaning, and doubt (Bryant and Astin, 2008).

It is important to note that spiritual struggles are not always detrimental. Rather, in some cases, spiritual struggles can promote wellbeing through their effect on meaning making, spiritual growth, and experiences of sacredness (Wilt et al., 2019; Zarzycka and Zietek, 2019; Zarzycka and Puchalska-Wasyl, 2020). Some scholars, including Wong (2019, 2020), have argued that struggling and flourishing are interdependent and that flourishing requires struggle. However, greater investigation is needed of the conditions under which this will occur or the factors that might buffer or exacerbate the effects of religious struggles on psychological distress. For example, Trevino et al. (2019) found that certain spiritual (insecure God attachment), social (social isolation), emotional (anger, death anxiety), and behavioral (smoking) burdens magnified the association between stressful life events and spiritual struggles; in contrast, spiritual (religious hope), social (emotional support), and cognitive (self-esteem, optimism) resources did not buffer effects. Additionally, Dworsky et al. (2016) found that, in addition to increased distress and negative psychological outcomes of spiritual struggles themselves, avoidance of spiritual struggles was associated with a multitude of mental health and emotional regulation difficulties. Together, these findings demonstrate the importance of evaluating potential moderators of spiritual struggles.

### The Relationship Between Spiritual Struggles and Closeness to God

Closeness to God and spiritual struggles generally have opposite associations with wellbeing, but the two are not necessarily orthogonal. It seems quite possible that a person could both feel quite close to God and be struggling spiritually at the same time. Just as closeness and perceived partner understanding buffers against deleterious effects of conflict in human relationships (Gordon and Chen, 2016), it may be that closeness to God buffers against the negative effects of spiritual struggles on wellbeing. Trevino et al. (2019) found that the related concept of insecure attachment to God exacerbated the effects of stressful life events on spiritual struggles, and previous research suggests other aspects of religiosity (i.e., religious commitment, life sanctification, religious support, religious hope) buffer the links between spiritual struggles and two outcomes: happiness and depressive symptoms (Abu-Raiya et al., 2016). However, such buffering effects have not been tested for closeness to God nor have effects been tested among a wide range of outcomes.

To date, the literature on closeness to God and spiritual struggles have largely examined these two factors separately. The few studies that do assess them both often do not examine or fully discuss the relationships between them. For example, Exline et al. (2000) found spiritual struggles positively predicted depressive symptoms and suicidality in college undergraduates even after controlling for religiosity and for religious comfort (a variable which shares some similarities to closeness to God); however, they did not report whether religious comfort was still a significant predictor after controlling for struggles. Likewise, Harris et al. (2014) examined the effects of both spiritual struggles and religious comfort on trauma symptoms, but they did not run a model including both as simultaneous predictors. In a later study, they found that religious comfort and particular types of spiritual struggles simultaneously predicted with postconventional religiousness, a mature religiousness characterized by a commitment to faith after addressing challenges and doubt (Harris et al., 2015). However, they did not report whether comfort and struggle both predicted wellbeing nor did they examine interactions between them.

With regard specifically to wellbeing, Abernethy et al. (2020) found that among inpatient psychiatric patients, religious comfort and spiritual struggles, as assessed by the Religious Comfort and Strain Scale (Exline et al., 2000, 2014), were moderately negatively correlated with each other and depressive symptoms, but only religious comfort predicted changes in depression from intake to discharge. Similarly, Abu-Raiya et al. (2015a,b) tested whether closeness to God predicted spiritual struggles in a sample of Israeli–Palestinian Muslim college students. Although the correlational analyses from this study revealed a significant, negative association between closeness to God and spiritual struggles (i.e., greater closeness to God was associated with less spiritual struggles), researchers found that closeness to God did not predict any type of spiritual struggles when they conducted hierarchical regression analyses controlling for God image, fundamentalism, and universality. Both closeness to God and spiritual struggles were simultaneous predictors of life satisfaction, but only spiritual struggles predicted depressive and anxiety symptoms. Moreover, they did not test for any interaction effects. Thus, examination of closeness to God, spiritual struggles, and their interaction in a large sample of diverse undergrads across the United States is needed.

### Spirituality and Belonging in College

Given our interest in college students, we expanded our focus on wellbeing to consider students’ belonging on campus. A person’s sense of belonging is the extent to which they feel comfortable, valued, and a part of a particular environment (sometimes called belongingness; Osterman, 2000; Walton and Brady, 2017). Past research has argued that belonging is not merely beneficial but, rather, a fundamental human need (Baumeister and Leary, 1995). A related, but distinct, construct is belonging uncertainty (Walton and Cohen, 2007). This is the extent to which a person is uncertain or unsettled about their belonging. Belonging uncertainty differs from belonging in that it captures the extent of a person’s worry about whether they belong rather than their level of belonging (Walton and Brady, 2017). Understanding how spiritual factors relate to belonging is critical because students’ perceptions and experiences of belonging early in college can affect their academic outcomes and wellbeing for years (Walton and Cohen, 2011; Brady et al., 2020).

A person’s sense of belonging is shaped by an understanding of their own social identity and how that identity “fits”—or does not—in their present context. For example, past studies demonstrate that a students’ gender, ethnicity, and status as a first-generation college student can affect how they perceive their environments and the extent to which they feel like they belong (Walton and Cohen, 2011; Stephens et al., 2014; Walton et al., 2015). To the extent that spirituality is an important aspect of identity, we expect that it, too, would affect students’ sense of belonging at college. However, little is known about the associations between spiritual variables and college students’ feelings of belonging.

We hypothesize that both closeness to God and spiritual struggles will be associated with belonging. A student who feels close to God may experience that relationship as a secure base from which to form attachments to other people on campus. They may have an easier time connecting with their faith community. The security they feel in their relationship with God may temper their concerns about belonging on campus. In contrast, a student who is struggling spiritually may have a hard time feeling connected and valued by others on campus. Spiritual struggles may not only lower their sense of belonging but also unsettle it. Relatedly, the feelings of anxiety or depression that can be brought on by spiritual struggles may make it more difficult to engage with others and foster a sense of belonging within a new community. No study, to our knowledge, has yet explored these important questions connecting belonging to aspects of spiritual identity.

### Hypotheses

The manuscript examines two key aspects of spirituality—closeness to God and spiritual struggles—of first-year college students across six different campuses in the United States. Our preregistered analyses, which are “underutilized in the psychology of religion/spirituality field,”^1^ examine the associations between these two factors and various indices of wellbeing. We hypothesize that students’ closeness to God will be positively related to better wellbeing, mental health, physical health, and college belonging and that spiritual struggles will be negatively associated with these same outcomes when simultaneously entered as predictors, even after accounting for demographic variables. See Table 1 for specific predictions related to each study variable and the literature related to those predictions. To complement general wellbeing measures, we assess an important college-specific indicator of wellbeing: students’ sense of belonging in college, to complement general wellbeing measures. We also examine interactions between closeness to God and spiritual struggles, exploring the possibility that closeness to God may be a protective factor that moderates the relation between spiritual struggles and negative outcomes.

**Table 1:** Hypotheses based on previous literature at time of preregistration.

Key variables	Reference studies	Hypothesized association for closeness to God	Reference studies	Hypothesized association for spiritual struggles
Life Satisfaction	Poloma and Pendleton, 1990 ^†^	Positive	Ellison, 1991; Ellison and Lee, 2010; Abu-Raiya et al., 2015a,b^†^	Negative
Happiness	Poloma and Pendleton, 1990 ^†^	Positive	Ellison, 1991; Ellison and Lee, 2010; Abu-Raiya et al., 2015a,b^†^	Negative
Meaning in Life	Poloma and Pendleton, 1990 ^†^	Positive	Exline and Rose, 2005; Park, 2005; Exline, 2013	Negative
Anxiety	Rowatt and Kirkpatrick, 2002; Boelens et al., 2012	Negative	Ano and Vasconcelles, 2005 ^†^; McConnell et al., 2006 ^†^; Abernethy et al., 2020	Positive
Depression	Maton, 1989; Exline et al., 2000 ^†^; Abernethy et al., 2020	Negative	Exline et al., 2000 ^†^; Ano and Vasconcelles, 2005 ^†^; McConnell et al., 2006 ^†^	Positive
Loneliness	Kirkpatrick et al., 1999 ^†^	Negative	Exline, 2013; McConnell et al., 2006	Positive
General Mental Health	Krause, 1998; Hill and Pargament, 2003	Positive	Ano and Vasconcelles, 2005; McConnell et al., 2006	Negative
Primary Stress Appraisal	Koenig et al., 1998; Tix and Frazier, 1998; Smith et al., 2000; Hill and Pargament, 2003	Negative	Exline et al., 2000; Pearce et al., 2006; Herrera et al., 2009	Positive
Secondary Stress Appraisal	Kirkpatrick, 1995; Hill and Pargament, 2003	Positive	Pearce et al., 2006; Herrera et al., 2009	Negative
Physical Health	Krause, 1998; Hill and Pargament, 2003	Positive	Koenig et al., 1998 ^†^	Negative
Belonging	Poloma and Pendleton, 1990; Osterman, 2000; Hill and Pargament, 2003	Positive	Osterman, 2000; Exline and Rose, 2005; Bryant and Astin, 2008	Negative
Belonging Uncertainty	Poloma and Pendleton, 1990; Osterman, 2000; Hill and Pargament, 2003	Negative	Walton and Cohen, 2007	Positive

† *A study that directly examined correlations between spiritual struggles and the variable of interest. For all other studies, the hypothesized results were based on extrapolation and theory*.

## Materials and Methods

### Study Context

The data for this study were collected as part of a large research project conducted at more than 20 colleges and universities in the United States and Canada during academic years 2015–2016 and 2016–2017 (Blodorn et al., 2018). The primary purpose of the project was to conduct a randomized controlled trial of a social-belonging intervention (Walton and Cohen, 2011; Walton et al., 2021, unpublished),^2^ which used stories and a written response to convey that challenges in the transition to college are normative and not a sign of non-belonging. However, the project also provided the opportunity to collect data for additional investigations, of which the present study was the only one focused on spirituality (for other manuscripts that use data from the larger project, see Carter et al., 2019; Holmes et al., 2019; LaCosse et al., 2020; Logel et al., 2020). We collected the data for the present study as part of a survey during the spring semesters of students’ first year (i.e., spring semester 2016 and 2017). The questions about closeness to God and spiritual struggles were collected at six institutions in the US (four non-sectarian private liberal arts colleges in the Midwest, one religiously affiliated liberal arts college in the Midwest, and one public university in the South). A technical summary of the larger project is available in the Supplementary Material. The Supplementary Material also includes information about sample size determination, all data exclusions, all manipulations, and all measures in the study. All participants were consented per Institutional Review Board approved procedures.

### Participants

In total, 1,190 college students from the six schools responded to the questions for the present study. Because our focus was on closeness to God, a scale that requires some degree of relationship to a deity or higher power of some kind, we retained only those respondents who reported an affiliation with a religious or spiritual group and who noted a belief in God, gods, or a higher power. Oviedo (2019) argues that religious meaning may operate differently for people who view it as a more central component of identity such that it “is not a kind of ‘universal pattern,’ but a variable that registers different levels of intensity and influence among a broad sample of population” (p. 38). Given this, we examine these associations only among students who self-identify as spiritual and/or belonging to a religious group. Although some students who identify as Agnostic, Atheist, or not Spiritual or Religious may have spiritual struggles (Exline et al., 2014) or other experiences of transcendence (Callaway et al., 2020), it is likely that their spiritual experiences are qualitatively distinct from those who identify as spiritual and/or religious and are therefore beyond the scope of this study.

The resulting analytic sample included 839 students, although not all students had data for every variable. Hereafter, we refer only to the students who were included in the analytic sample. Most of the students (*n* = 762) were Christian, including those who identified as Protestant (59%), Catholic (29%), Christian Orthodox (2%), Mormon (<1%), and Jehovah’s Witness (<1%). Non-Christian students (*n* = 77) composed the remainder of the sample, including students who identified as Spiritual but not Religious (5%), Jewish (1%), Muslim (1%), Buddhist (1%), Hindu (<1%), Unitarian (<1%), Baha’i (<1%), Taoist (<1%), and Wiccan (<1%).

Overall, 63% of students were women, 37% of students were men, and less than 1% identified in another way regarding gender. Regarding race/ethnicity, the majority of students identified as White (72%), and a minority identified as Black (10%), Latino (7%), Asian (3%), Multiracial (4%), Native American (<1%), Other (3%), or did not provide a response (<1%). Twenty-seven percent were first-generation college students (i.e., no parent/guardian had earned a four-year college degree). For some analyses, we included indicator variables for whether a student was from an underrepresented racial/ethnic background (URM; Native, Black, and Latinx students; *n* = 156) and for whether students were a first-generation college student (*n* = 200).

### Procedure

As noted above, the questions for the present study were part of a survey students completed toward the end of their first year of college. The survey was administered online. In addition to the questions discussed here, students answered other questions about their social and academic experiences at college. Students received a $5 Amazon.com gift card for completing the 30-min survey.

Due to the risk of losing participants if the survey was overly long, the research team took a “practical measurement” approach (Duckworth and Yeager, 2015), assessing each of the constructs of interest with a brief measure, sometimes as short as a single question. Single-item measures of psychological variables are a viable alternative in education settings when testing time is limited (e.g., they reproduce theoretical underpinnings of long-form scales, correlate adequately with long-form scales; Gogol et al., 2014). Likewise, single-item measures of religiosity have been found to have adequate reliability and validity in student samples (Dollinger and Malmquist, 2009). Due to data use agreements with participating schools, the data cannot be made available in a public repository. However, data can be accessed by contacting the College Transition Collaborative (contact@ctcteam.org) and signing a data use agreement.

### Measures

#### Spiritual Variables

##### Closeness to God

Students reported their perceived closeness to a higher power ranging from 1 (*not at* all) to 5 (*extremely close*^3^) on an item adapted from the Daily Spiritual Experiences Scale (Underwood and Teresi, 2002): “Over the past month of school, how close have you felt to God, gods, or a higher power?”

##### Spiritual Struggles

Students reported the extent to which they recently have experienced spiritual struggles on one item adapted from the Religious and Spiritual Struggles Scale (Exline et al., 2014): “In the past month of school, how often have you had struggles related to religion or spirituality? Struggles can include feelings of confusion or doubt about your religious/spiritual beliefs; feeling as though your life had no deeper meaning; conflicts with other people about religious/spiritual matters or feeling hurt/mistreated by religious people; or feeling attacked by evil force” (scale: *1 = never/not at all* to *5 = very often*).

#### Wellbeing

##### Happiness

Students answered two questions from the Subjective Happiness Scale (Lyubomirsky and Lepper, 1999): “Compared with most of my peers, I consider myself …” (scale: 1 = *less happy* to 7 = *more happy*) and “In general, I consider myself …” (scale: 1 = *not a very happy person* to 7 = *a very happy person* scale).

##### Life Satisfaction

Students answered one question about life satisfaction (adapted from Andrews and McKennell, 1980): “All things considered, how satisfied are you with your life as a whole?” (scale: 1 = *totally dissatisfied* to 10 = *totally satisfied*).

##### Meaning in Life

Students answered one question about the extent to which they felt their life had meaning (adapted from Fredrickson et al., 2013): “Right now, how much do you feel that your life at [school name] has a sense of direction or meaning to it?” (scale: 1 = *not at all* to 5 = *a great deal*).

#### Mental Health

##### Anxiety

Students completed a two-question screener for anxiety (the GAD-2; Kroenke et al., 2009): “Over the last 2 weeks, how often have you been bothered by the following problems? (1) Not being able to stop or control worrying, (2) Feeling nervous, anxious, or on edge” (scale: 1 = *not at all* to 4 = *nearly every day*).

##### Depression

Students completed a two-question screener for depression (the PHQ-2; Kroenke et al., 2009): “Over the last 2 weeks, how often have you been bothered by the following problems? (1) Feeling down, depressed, or hopeless, (2) Little interest or pleasure in doing things” (scale: 1 = *not at all* to 4 = *nearly every day*).

##### Loneliness

Students answered two questions from the Three-Item Loneliness Scale (Hughes et al., 2004): “At [school name], how often do you feel isolated from others?” and “At [school name], how often do you feel that you lack companionship?” (scale: 1 = *hardly ever* to 3 = *often*).

##### General Mental Health

Students answered one question about their general mental health (adapted from a similar item about physical health, Ware and Sherbourne, 1992): “In general, how has your mental health been this past academic year?” (scale: 1 = *poor* to 5 = *excellent*).

#### Stress and Physical Health

##### Primary Appraisal of Stress

Students answered one question about how much stress they experience at college (modeled after Lazarus, 1991): “How much stress do you experience on a day-to-day basis at [school name]?” (scale: 1 = *none* to 7 = *an extreme amount*).

##### Secondary Appraisal of Stress

Students answered one question about how equipped they feel to cope with daily stress (modeled after Lazarus, 1991): “How confident do you feel that you can handle the stress you experience on a day-to-day basis at [school name]?” (scale: 1 = *not at all confident* to 7 = *extremely confident*).

##### Physical Health

Students answered one question about their physical health (Ware and Sherbourne, 1992): “In general, how has your physical health been this past academic year?” (scale: 1 = *poor* to 5 = *excellent*).

#### Belonging at College

##### Belonging

Students answered four questions about belonging in college (Walton and Cohen, 2007; for example, “I feel like I belong at [school name]”; scale: 1 = *strongly disagree* to 6 = *strongly agree*).

##### Belonging Uncertainty

Students answered one question about the extent to which they feel uncertain about their belonging at college (Yeager et al., 2013): “When you think about [school name], how often, if ever, do you wonder: ‘Maybe I do not belong here’?” (scale: 1 = *never* to 5 = *always*).

### Analysis Strategy

After calculating correlation coefficients and descriptives, we used R (R Core Team, 2017) and the lme4 package (Bates et al., 2015) to examine the associations between the spiritual variables (closeness to God and spiritual struggles) and wellbeing, mental health, stress and physical health, and belonging variables. Each model included both of the spiritual variables, in order to assess unique variance of each in relation to the dependent variables. In each model, as specified in our preregistration,^4^ we included gender (1 = female, 0 = male; three missing) and intervention treatment as covariates. In addition, we included dummy variables for three additional demographic variables of interest: Christian affiliation (1 = Christian, 0 = Non-Christian), URM background (1 = Black, Latinx, and/or Native, 0 = Not Black, Latinx, or Native; one missing), and first-generation college (1 = First-generation college, 0 = Not first-generation college; 56 missing).^5^ We also included in each model a random intercept for school to account for differences across institutions. To generate *p*-values, we used the lmerTest package (Kuznetsova et al., 2014). Participants were dropped for a particular analysis if they had missing data for any of the variables in that analysis; that said, all analyses included at least 92% of participants. We also conducted exploratory analyses testing for moderation of closeness to God on spiritual struggle using the R interactions package for simple slopes analyses (Long, 2019).

## Results

### Preliminary Analyses

Table 2 provides descriptive statistics and correlations for the key variables. Although we used a modified, shorter scale to measure spiritual struggles, participants’ scores were similar to those observed in other studies (McConnell et al., 2006; Abu-Raiya et al., 2015a,b). Similarly, closeness to God scores were similar to those observed in past research (e.g., Underwood and Teresi, 2002).

**Table 2:** Zero-order correlations.

	1	2	3	4	5	6	7	8	9	10	11	12	13	14	15
1. Closeness to God	–														
2. Spiritual Struggles	−0.15^***^	–													
3. Happiness	0.26^***^	−0.20^***^	–												
4. Life Satisfaction	0.26^***^	−0.20^***^	0.64^***^	–											
5. Meaning in Life	0.25^***^	−0.10^***^	0.43^***^	0.56^***^	–										
6. Anxiety	−0.06^*^	0.16^***^	−0.32^***^	−0.34^***^	−0.23^***^	–									
7. Depression	−0.15^***^	0.21^***^	−0.44^***^	−0.49^***^	−0.38^***^	0.60^***^	–								
8. Loneliness	−0.11^***^	0.13^***^	−0.37^***^	−0.42^***^	−0.43^***^	0.33^***^	0.43^***^	–							
9. Mental Health	0.19^***^	−0.18^***^	0.48^***^	0.50^***^	0.39^***^	−0.52^***^	−0.54^***^	−0.38^***^	–						
10. Primary Stress Appraisal	−0.05	0.15^***^	−0.25^***^	−0.30^***^	−0.21^***^	0.57^***^	0.41^***^	0.24^***^	−0.48^***^	–					
11. Secondary Stress Appraisal	0.20^***^	−0.11^***^	0.39^***^	0.46^***^	0.36^***^	−0.46^***^	−0.41^***^	−0.27^***^	0.52^***^	−0.40^***^	–				
12. Physical Health	0.18^***^	−0.14^***^	0.26^***^	0.30^***^	0.21^***^	−0.25^***^	−0.25^***^	−0.20^***^	0.48^***^	−0.26^***^	0.33^***^	–			
13. Belonging	0.13^***^	−0.08^**^	0.37^***^	0.49^***^	0.55^***^	−0.24^***^	−0.36^***^	−0.59^***^	0.38^***^	−0.21^***^	0.33^***^	0.18^***^	–		
14. Belonging Uncertainty	−0.15^***^	0.12^***^	−0.34^***^	−0.46^***^	−0.52^***^	0.27^***^	0.37^***^	0.54^***^	−0.37^***^	0.24^***^	−0.33^***^	−0.17^***^	−0.71^***^	–	
15. Intervention Treatment	−0.05	0.03	−0.03	−0.01	0.01	−0.02	0.03	−0.00	0.01	−0.01	−0.01	0.02	0.01	0.00	–
Mean	2.89	2.41	5.33	7.35	3.67	2.15	1.72	1.59	2.96	4.29	4.52	3.31	4.89	2.27	
SD	1.26	1.11	1.22	1.88	1.09	0.93	0.83	0.61	1.12	1.31	1.39	1.10	0.97	0.93	
*N*	839	839	834	834	834	838	838	839	839	839	839	839	839	839	
Range	4	4	6	9	4	3	3	2	4	6	6	4	5	4	

* *p* *< 0.05;*

** *p* *< 0.01;*

*** *p < 0.001*.

There was meaningful variability in the spiritual variables across different campuses for both closeness to God, *F*(5, 833) = 14.64, *p* < 0.001, *η^2^* = 0.08, and spiritual struggles, *F*(5, 833) = 3.18, *p* < 0.01, *η^2^* = 0.02. Students at a large public university in the southeast reported the highest average closeness to God (*M* = 3.31, SD = 1.25), whereas students at one of the private liberal arts colleges reported average closeness to God that was almost a full point lower (*M* = 2.38, SD = 1.23). Students at a faith-based liberal arts college reported the highest average spiritual struggles (*M* = 2.67, SD = 1.06), whereas students at a different liberal arts college reported the lowest average spiritual struggles (*M* = 2.23, SD = 0.97).

Table 2 presents the zero-order correlations among the key variables. Of note, the two spiritual variables are significantly, but not strongly, negatively correlated (*r* = −0.15, *p* < 0.001). Because closeness to God and spiritual struggles were hypothesized to predict the same outcomes but in opposite directions, some degree of negative correlation was expected between the two variables. However, the small association suggests that they are not redundant.

### Preregistered Analyses

Across all significant results, effects were small to moderate in size.

#### Do Closeness to God and Spiritual Struggles Relate to Wellbeing?

After accounting for the covariates, closeness to God was positively associated with all three indicators of wellbeing: happiness, life satisfaction, and meaning in life. Spiritual struggles negatively associated with happiness, life satisfaction, and meaning in life. In addition, Christian students reported higher happiness, and URM students reported lower life satisfaction and meaning in life (see Table 3).

**Table 3:** Wellbeing variables regressed on closeness to god and spiritual struggles, controlling for demographic variables, and treatment condition.

	Happiness			Life satisfaction			Meaning in life		
	*β*	*b* (SE)	*p*	*β*	*b* (SE)	*p*	*β*	*b* (SE)	*p*
Closeness to god	0.220	0.218 (0.026)	<0.001^***^	0.241	0.352 (0.038)	<0.001^***^	0.228	0.190 (0.022)	<0.001^***^
Spiritual struggles	−0.183	−0.207 (0.029)	<0.001^***^	−0.185	−0.308 (0.043)	<0.001^***^	−0.082	−0.078 (0.025)	0.002^**^
Christian	0.079	0.340 (0.110)	0.002^**^	0.014	0.088 (0.162)	0.590	−0.029	−0.104 (0.095)	0.277
Gender (Female = 1)	−0.011	−0.028 (0.067)	0.673	−0.032	−0.123 (0.098)	0.212	0.013	0.030 (0.059)	0.616
URM background	−0.021	−0.068 (0.087)	0.436	−0.089	−0.432 (0.128)	0.001^**^	−0.090	−0.250 (0.075)	0.001^**^
First-generation college	−0.026	−0.072 (0.073)	0.321	−0.041	−0.168 (0.107)	0.117	−0.038	−0.088 (0.063)	0.165
Intervention treatment	0.003	0.007 (0.068)	0.918	0.018	0.069 (0.1)	0.486	0.025	0.057 (0.058)	0.328

** *p* *< 0.01;*

*** *p < 0.001*.

#### Do Closeness to God and Spiritual Struggles Relate to Mental Health?

As predicted, closeness to God was negatively associated with anxiety, depression, and loneliness, and positively associated with general mental health. The patterns were opposite for spiritual struggles. Spiritual struggles were positively associated with anxiety, depression, and loneliness, and negatively associated with general mental health. In addition, URM students reported greater depression and loneliness and lower mental health. First-generation college students reported greater depression (see Table 4).

**Table 4:** Mental health variables regressed on closeness to god and spiritual struggles, controlling for demographic variables and treatment condition.

	Anxiety			Depression			Loneliness			Mental health		
	*β*	*b* (SE)	*p*	*β*	*b* (SE)	*p*	*β*	*b* (SE)	*p*	*β*	*b* (SE)	*p*
Closeness to god	−0.041	−0.029 (0.019)	0.134	−0.122	−0.079 (0.017)	<0.001^***^	−0.085	−0.041 (0.013)	0.002^**^	0.172	0.149 (0.023)	<0.001^***^
Spiritual struggles	0.160	0.131 (0.021)	<0.001^***^	0.214	0.157 (0.019)	<0.001^***^	0.117	0.064 (0.015)	<0.001^***^	−0.172	−0.170 (0.025)	<0.001^***^
Christian	−0.042	−0.130 (0.083)	0.114	−0.048	−0.135 (0.074)	0.067	−0.058	−0.123 (0.056)	0.028^*^	0.024	0.091 (0.097)	0.348
Gender (Female = 1)	0.191	0.363 (0.052)	<0.001^***^	0.053	0.091 (0.047)	0.053	0.108	0.138 (0.034)	<0.001^***^	−0.171	−0.395 (0.059)	<0.001^***^
URM background	−0.026	−0.062 (0.064)	0.338	0.073	0.156 (0.057)	0.006^**^	0.088	0.142 (0.044)	0.001^**^	−0.056	−0.161 (0.076)	0.036^*^
First-generation college	0.021	0.043 (0.055)	0.436	0.069	0.123 (0.049)	0.012^*^	0.050	0.067 (0.037)	0.069	0.014	0.034 (0.064)	0.593
Intervention treatment	−0.041	−0.080 (0.050)	0.107	0.000	0.000 (0.044)	0.992	−0.013	−0.017 (0.034)	0.611	0.023	0.053 (0.059)	0.371

* *p* *< 0.05;*

** *p* *< 0.01;*

*** *p < 0.001*.

#### Do Closeness to God and Spiritual Struggles Relate to Stress and Physical Health?

Closeness to God and spiritual struggles were associated with some but not all of the stress and physical health variables. Specifically, closeness to God was associated with two positive outcomes: secondary stress appraisal (i.e., ability to cope with stress) and physical health, but not primary stress appraisal. In contrast, spiritual struggles were associated with only primary stress appraisal (i.e., how much stress). In addition, women reported worse physical health, higher primary stress, and lower secondary stress. URM students reported worse physical health but lower primary stress (see Table 5).

**Table 5:** Stress and physical health outcomes regressed on closeness to god and spiritual struggles, controlling for demographic variables and treatment condition.

	Primary stress			Secondary stress			Physical health		
	*β*	*b* (SE)	*p*	*β*	*b* (SE)	*p*	*β*	*b* (SE)	*p*
Closeness to god	−0.035	−0.036 (0.028)	0.199	0.213	0.231 (0.028)	<0.001^***^	0.184	0.157 (0.023)	<0.001^***^
Spiritual struggles	0.155	0.18 (0.031)	<0.001^***^	−0.072	−0.089 (0.032)	0.005^**^	−0.109	−0.106 (0.025)	<0.001^***^
Christian	0.008	0.035 (0.118)	0.764	0.006	0.027 (0.122)	0.826	−0.004	−0.015 (0.097)	0.877
Gender (Female = 1)	0.137	0.369 (0.073)	<0.001^***^	−0.204	−0.589 (0.074)	<0.001^***^	−0.184	−0.417 (0.062)	<0.001^***^
URM background	−0.056	−0.188 (0.092)	0.042^*^	−0.017	−0.061 (0.096)	0.524	−0.072	−0.204 (0.075)	0.007^**^
First-generation college	0.045	0.126 (0.078)	0.105	−0.048	−0.146 (0.08)	0.070	0.001	0.003 (0.064)	0.964
Intervention treatment	−0.014	−0.039 (0.071)	0.584	0.005	0.016 (0.075)	0.833	0.043	0.099 (0.058)	0.091

* *p* *< 0.05;*

** *p* *< 0.01;*

*** *p < 0.001*.

#### Do Closeness to God and Spiritual Struggles Relate to Belonging and Belonging Uncertainty?

As predicted, closeness to God was positively associated with belonging and negatively associated with belonging uncertainty. In contrast, spiritual struggles were positively associated with belonging uncertainty, but not significantly associated with belonging. URM students reported lower belonging and higher belonging uncertainty than their peers did. For first-generation students, the same pattern was evident, albeit weaker and not statistically significant (see Table 6).

**Table 6:** Belonging variables regressed on closeness to god and spiritual struggles, controlling for demographic variables and treatment condition.

	Belonging			Belonging Uncertainty		
	*β*	*b* (SE)	*p*	*β*	*b* (SE)	*p*
Closeness to god	0.098	0.074 (0.021)	<0.001^***^	−0.102	−0.076 (0.02)	<0.001^***^
Spiritual struggles	−0.077	−0.067 (0.023)	0.004^**^	0.110	0.094 (0.022)	<0.001^***^
Christian	−0.007	−0.024 (0.088)	0.783	−0.007	−0.023 (0.087)	0.794
Gender (Female = 1)	−0.044	−0.090 (0.056)	0.110	0.023	0.046 (0.055)	0.401
URM background	−0.168	−0.425 (0.069)	<0.001^***^	0.141	0.350 (0.067)	<0.001^***^
First-generation college	−0.042	−0.089 (0.059)	0.126	0.034	0.070 (0.057)	0.225
Intervention treatment	0.018	0.036 (0.053)	0.492	−0.006	−0.013 (0.052)	0.802

** *p* *< 0.01;*

*** *p < 0.001*.

### Exploratory Analyses: Is Closeness to God a Protective Factor Between Spiritual Struggles and Negative Outcomes?

Although closeness to God and spiritual struggles generally had opposing associations with our outcome variables, their zero-order correlation was quite small, suggesting the possibility that they functioned relatively independently. Thus, on an exploratory basis, we also explored whether closeness to God and spiritual struggles interacted to predict wellbeing outcomes. In addition to the R analysis packages described above, we used the interactions package for simple slopes analyses (Long, 2019).

For most of our outcome variables, we did not observe significant interactions between closeness to God and spiritual struggles. However, there were two exceptions to this: depression and loneliness (see Table 7). Simple slopes analyses suggested that closeness to God may buffer the association between spiritual struggles and negative psychological outcomes. As shown in Table 8, students with high closeness to God (+1 SD), spiritual struggles were not significantly correlated with loneliness; in contrast, for those at or below the mean on closeness to God, spiritual struggles were significantly and positively associated with loneliness. A similar attenuation effect was observed for depression; even though spiritual struggles and depression were significantly associated at all levels of closeness to God, the effect size for this association was smaller at higher levels of closeness to God.

**Table 7:** Depression and loneliness regressed on closeness to god, spiritual struggles, and their interaction.

	Depression			Loneliness		
	*β*	*b* (SE)	*p*	*β*	*b* (SE)	*p*
Closeness to god	−0.004	−0.002 (0.035)	0.948	0.042	0.021 (0.027)	0.442
Spiritual struggles	0.333	0.246 (0.041)	<0.001^***^	0.276	0.153 (0.031)	<0.001^***^
Intervention treatment	0.019	0.033 (0.042)	0.431	−0.010	−0.014 (0.032)	0.672
Spiritual struggles × Closeness to god	−0.176	−0.035 (0.014)	0.009^*^	−0.210	−0.031 (0.010)	0.002^**^

* *p* *< 0.05;*

** *p* *< 0.01;*

*** *p < 0.001*.

**Table 8:** Simple slopes of spiritual struggles at +1 SD, the Mean, and −1 SD of closeness to god.

Closeness to God	Depression		Loneliness	
	*b* (SE)	*p*	*b* (SE)	*p*
+1 SD	0.100 (0.026)	<0.001^***^	0.023 (0.02)	0.259
Mean	0.145 (0.018)	<0.001^***^	0.063 (0.014)	<0.001^***^
−1 SD	0.190 (0.024)	<0.001^***^	0.103 (0.018)	<0.001^***^

*** *p < 0.001*.

## Discussion

This study examined closeness to God and spiritual struggles among first-year college students who identified as religious or spiritual. First, we replicated previous effects of closeness to God and spiritual struggle on wellbeing outcomes in a preregistered, large sample. Although these elements of our research may not be entirely novel, they contribute to a robust and incremental science. Leading scholars in psychology of religion and spirituality argue that large-scale replication projects are necessary to ensure the credibility of the field (Van Elk et al., 2015; Van Elk, 2019). Additionally, we make two novel contributions. We showed that closeness to God was positively associated with belonging and negatively associated with belonging uncertainty, relationships that, to our knowledge, have not been examined in previous research, especially in a time of important transition. Additionally, we found potential buffering effects of closeness to God on the deleterious effects of spiritual struggles.

For basic replications of previous findings on closeness to God, hypothesized correlations were observed for 11 out of 12 outcomes (with primary stress appraisal as the exception). Likewise, significant associations were found for 8/12 outcomes for spiritual struggles; we did not find support for the hypothesized negative relationships with meaning in life, secondary stress appraisal, physical health, or belonging. Taken together, these results suggest general replicability of findings, but they also suggest further work is needed regarding the generalizability across diverse populations for spiritual struggles.

### Simultaneous Effects of Closeness to God and Spiritual Struggles

Results suggest that some effects for closeness to God and spiritual struggles were additive for some outcome variables but would wash out for others. For seven out of 12 outcome variables, significant associations were observed for both closeness to God and spiritual struggles when the two were simultaneously entered in a regression model. However, for five out of 12 outcomes, only one of the spirituality variables was significant when both were included in the model. Among the stress and physical health variables, only closeness to God was associated with positive outcomes of secondary stress appraisal and physical health, and only spiritual struggles were associated with primary stress appraisal, generally considered to be a negative outcome. These findings may point to critical differences between the predictors such that closeness to God tends to serve as a source of comfort or coping whereas spiritual struggles serve as a direct stressor. These findings are notable in that nearly all the extant literature examines only one of these variables at a time, and few studies have examined their concurrent effects.

Among wellbeing outcomes, our results indicated that spiritual struggles were not associated with meaning in life—unlike closeness to God, which was positively associated. The lack of a significant association may be due to differences in types of spiritual struggle that may not be adequately captured in a one-item measure. For example, some types of spiritual struggle may result in a crisis of meaning (e.g., “What if everything I’ve been taught to believe is wrong?”), but other types of spiritual struggle may eventually lead to greater clarity and potentially resolution and growth (e.g., “I’m currently unsure of what I believe, but I know I can figure it out”; Exline and Rose, 2005; Desai and Pargament, 2015). The differences in associations between closeness to God and spiritual struggles when predicting key outcomes suggest longitudinal studies assessing the dynamics of these variables across time are warranted.

Finally, our results show that closeness to God was associated with both belonging and belonging uncertainty for religious students, but spiritual struggles were only significantly associated with belonging uncertainty. Because closeness to God is, at its core, a variable dealing with relational connectedness, it would make sense that it would be associated with both belonging and belonging uncertainty. Spiritual struggles, on the other hand, encompasses many different types of stressors dealing with spirituality that would be more likely to increase isolation, lead to social disconnect, and increase belonging uncertainty but might not necessarily affect feelings of belonging for religious college students. This is the first study to examine how closeness to God and spiritual struggles are associated with belonging variables, so these findings should be replicated before firm conclusions are drawn.

### Closeness to God Attenuates Some Negative Effects of Spiritual Struggles

In our exploratory analyses testing interactions between closeness to God and spiritual struggles among students who identify as religious, we found that when participants were higher in closeness to God, spiritual struggles were less strongly associated with depression and were no longer associated with loneliness. However, this attenuation was not found for any positive outcomes, and it was not consistent across most negative outcomes. These results suggest that closeness to God may help avert some negative outcomes of spiritual struggles, which are common among college students as they encounter challenges to their faith in new environments (Bryant and Astin, 2008).

These exploratory findings extend existing findings in both the religious coping and spiritual struggles literature (Krause, 1998; Pargament et al., 1998; Tix and Frazier, 1998; Bryant and Astin, 2008; Laurin et al., 2014; Jeppsen et al., 2015; Trevino et al., 2019; Wilt et al., 2019; Zarzycka and Zietek, 2019; Zarzycka and Puchalska-Wasyl, 2020). Although separate research on closeness to God (a protective factor) and spiritual struggles (a source of stress) indicates that the two variables have the potential to relate to one another, only a handful of studies examined them together (Exline et al., 2000; Harris et al., 2014; Abernethy et al., 2020) or using closely related constructs (Trevino et al., 2019). However, these findings are based on exploratory analyses, so more research is necessary before making strong conclusions.

### Practical Implications

This study highlights the importance of closeness to God and spiritual struggles for first-year students at colleges and universities in the United States who identify as spiritual or religious, providing support for the stance of Astin (2004) that matters of religion and spirituality should be considered an important aspect of student development. Although religiosity may shift or decline during the college years (Koenig et al., 2012; Barry and Abo-Zena, 2014), our findings suggest there are aspects of spirituality that continue to be associated with important outcomes, at least during students’ first year of college among the majority of students for whom spirituality or religion is a part of their life. Many schools work diligently to address the many challenges that accompany the first year of college through organized activities, academic programming, and psychological interventions with the goal of integration with the college environment (Tinto, 1987; Bohnert et al., 2007; Inkelas et al., 2007; Walton and Cohen, 2011; Arnett, 2014; Yeager et al., 2016). It would behoove college administrators to also pay greater attention to spirituality when trying to promote feelings of belonging and inclusion for all students during the first year of college. Dworsky et al. (2016) suggested therapists and counselors use Acceptance-Based Therapies (ACT) to mitigate the negative impacts of avoiding and pushing away spiritual struggles. Additionally, these findings suggest that university counseling center therapists or other student life personnel might recognize that spiritual struggles may or may not be related to negative outcomes, depending on the student’s closeness to God and the specific outcome.

### Constraints on Generality and Directions for Future Research

The generality of our findings is likely constrained to first-year students at four-year residential colleges in the United States who identify as religious or spiritual. Notably, our data were collected before the COVID-19 pandemic. Although it is unknown whether results would differ if data were collected during or after the pandemic, we anticipate the findings would remain similar, perhaps with magnified effect sizes. Other research suggests spiritual struggles were higher during the pandemic, but religious resources were protective for health and wellbeing outcomes during the pandemic (e.g., Coppola et al., 2021).

We excluded students who did not identify as religious or spiritual, and findings may differ for them. For example, they may show little to no variability on these variables. Alternately, spiritual struggles may not have similarly deleteriously effects, or closeness to God may not buffer against struggles. Just as the United States is primarily Christian, so too was our sample; we would caution against extension of findings to predominantly non-Christian contexts. For instance, Sharma et al. (2009) describe slightly different mechanisms by which spiritual practices more common in South Asia (i.e., yoga, meditation, and practices that open one to psychic realms) produce positive physical and mental health outcomes, including the mitigation of depression, anxiety, and schizophrenia symptoms. Moreover, the findings may not apply equally across all Christian denominations, which is an important variable to consider in future work. With those caveats, we anticipate that our findings would generally extend to a broad population of college students, including those past their first year, since the findings largely supported relationships observed in other studies of United States adults. That said, developmental changes may affect some of the relationships, particularly those associated with belonging.

There could be other contexts—potentially even other college contexts—in which spiritual struggles could have a neutral or even positive effect on the outcomes measured here. If people have a supportive spiritual community, with leaders or mentors with whom they can discuss their spiritual struggles, they could be encouraged to work through struggles and grow from them. In that context, they could experience those struggles as a normative and positive part of their journey toward closeness to God specifically or in their spiritual journey more generally.

As noted above, the data for the present research were collected as part of a larger study that tested the efficacy of a social-belonging intervention (see Walton et al., 2021, Unpublished; see footnote 2 for results of the main analysis looking at effects on structurally disadvantaged students; see also LaCosse et al., 2020 and Logel et al., 2020, for positive effects of the intervention on two other stigmatized groups). Although the intervention content did not relate to spirituality and our analyses included intervention condition as a covariate, future research should collect data from a similar sample of students for the sole purpose of examining spiritual variables and their associations with important college outcomes. A study of this nature would also permit the use of longer measures for the variables of interest, reducing measurement error. It would also provide the opportunity to replicate and better understand the exploratory interaction effects we found.

The cross-sectional nature of the data precludes inferences regarding the directionality of effects. Previous work with psychiatric inpatients found that religious comfort, which is akin to closeness to God, predicted subsequent depression in an autoregressive cross-lagged model, but spiritual struggles and depression were not significant cross-lagged predictors (Abernethy et al., 2020). Thus, it is possible that closeness to God and spiritual struggles might predict subsequent wellbeing, wellbeing might predict subsequent spirituality, or that these variables are only cross-sectionally correlated.

Experimental research that tests a causal link between closeness to God or spiritual struggles and college outcomes would clarify the directionality of effects and provide even more support for administrators to pursue activities and programming that cultivate students’ spiritual identities, at least for students who identify as religiously affiliated. The connection between belonging and spiritual variables may also provide a rich area to further examine in the future. Rios et al. (2015) have begun research in this area when they examined how religious identity may impact academic outcomes. If religious students’ sense of belonging could be better supported in academic environments where stereotype threat based on religious identity may arise (perhaps by supporting students’ sense of closeness to God), then academic outcomes for those students could be improved. Last, further research is necessary to explore the moderating effect of closeness to God and determine how such an effect could be utilized by university administrators.

## Conclusion

Our results highlight the importance of considering closeness to God and spiritual struggles for wellbeing, mental health, and belonging during the first year of college alongside other variables typically examined in higher education contexts. Additionally, our study provides greater insights into the role of closeness to God in buffering against the negative effects of spiritual struggles on negative outcomes for first-year college students during this critical period.

## Data Availability Statement

The data underlying the results presented in the study are available from the College Transition Collaborative (https://collegetransitioncollaborative.org/) *via* contact from CL (clogel@uwaterloo.ca).

## Ethics Statement

This secondary analysis of data was approved by the Human Subjects Review Committee at Fuller Theological Seminary (#a4/18.103). The participants provided their written informed consent to participate in this study.

## Author Contributions

MG, SB, SS, and CL: conceptualization and methodology. MG and SB: software, formal analysis, data curation, and visualization. SB: validation. CL: resources. MG, SS, and TA: writing—original draft preparation. SB, SS, and CL: writing—review and editing. SS and CL: supervision. MG and CL: project administration. CL: funding acquisition. All authors contributed to the article and approved the submitted version.

## Funding

This paper analyzes data from a larger dataset collected by the College Transition Collaborative testing the effects of prematriculation social-belonging interventions on students’ experience in the transition to college (PIs: Christine Logel, Mary Murphy, Greg Walton, and David Yeager). The CTC Belonging Project was funded by the Raikes Foundation, the National Science Foundation, the Higher Education Quality Council of Ontario, and Partner Schools, made possible through methods and data systems created by the Project for Education Research That Scales (PERTS), and supported by the Mindset Scholars Network.

## Conflict of Interest

The authors declare that the research was conducted in the absence of any commercial or financial relationships that could be construed as a potential conflict of interest.

## Publisher’s Note

All claims expressed in this article are solely those of the authors and do not necessarily represent those of their affiliated organizations, or those of the publisher, the editors and the reviewers. Any product that may be evaluated in this article, or claim that may be made by its manufacturer, is not guaranteed or endorsed by the publisher.

## Footnotes

^1^ https://www.openscienceofreligion.com/call-for-proposals

^2^Walton, G. M., Murphy, M. C., Logel, C., Yeager, D, and The College Transition Collaborative (2021). Where and with whom does a brief social-belonging intervention raise college achievement? [Unpublished manuscript]. The CTC Belonging Trial.

^3^Students also had the option to select “0 = N/A; I do not believe in a higher power.” As noted above, these students have been excluded from the present sample.

^4^We pre-registered our hypotheses and primary analyses on AsPredicted.org.

^5^We added these additional covariates to our-preregistered analytic models in response to feedback from colleagues and reviewers. The pre-registered models, which include both of the spiritual variables as well as controls for treatment condition and gender, yield largely the same results, as do simpler models that examine the spiritual variables separately while controlling for treatment and gender.
