# Supplementary material for: Closeness to God, Spiritual Struggles, and Wellbeing in the First Year of College
Source: Front Psychol. 2022 Mar 31;13:742265. doi: 10.3389/fpsyg.2022.742265 (PMC9008207; doi:10.3389/fpsyg.2022.742265)
Supplement: Supplementary file 1 [file Data_Sheet_1.PDF]

ONLINE SUPPLEMENTAL MATERIALS

Closeness to God, Spiritual Struggles, and Well-being in the Transition to College

Table of Contents

Description of the broader study 2

Intervention materials and survey items from the broader study 3

Follow-up survey from which data were pulled 63

Description of the Broader Study

Data were drawn from a larger study examining the effectiveness of a matriculation social belonging intervention to increase student well-being and academic outcomes among college freshmen in the US and Canada. The larger study sampled participants from 23 colleges/universities for a total of 31,232 participants. The data were collected by the College Transition Collaborative, (https://collegetransitioncollaborative.org/), which is a researcher-practitioner partnership that develops psychologically informed and evidence-based interventions to increase equity and inclusion in higher education settings.

Participants were recruited during freshmen orientation to engage in a 30-minute online activity to learn about what their school is like from upperclassmen. Participants were assigned to one of three conditions: a belonging intervention, a belonging intervention customized to the particular school, or an active control. After completing their assigned intervention, participants completed a follow-up survey.

During spring semester of their freshmen year, a random selection of participants from socially disadvantaged and advantaged groups completed a follow-up survey to assess their experiences during the first year as well as well-being. Data for the current study were drawn from this follow-up survey.

College Transition Collaborative Belonging Intervention Materials

Start of Block: Introduction

You’re in.

You're coming to [SCHOOL NAME].

You're excited.

You probably have lots of questions about what to expect. This activity will give you an opportunity to learn more about students’ experiences coming to [SCHOOL NAME]. Because we value your point of view, we’ll also ask you to share your thoughts and feelings about coming to [SCHOOL NAME].

We want to learn more about your and other students’ perspectives so we can give future [SCHOOL NAME] students better insight into what coming to college is like. Welcome to [SCHOOL NAME].

We're very glad you're (almost) here.

Page Break

Important Notes
You probably have many questions about what [SCHOOL NAME] will be like. These materials will give you the opportunity to learn more about, to reflect on, and to share your perspectives on the transition to [SCHOOL NAME].

This activity will take approximately 30 minutes. Please do it in one sitting, on your own, in a quiet and private setting with your full attention and engagement.

If you don’t think you can complete this without interruption for the next 30 minutes or so, please close the page now and do it at another time when you can complete it in a single session.

Thank you!

Page Break

On some pages, the "next" button will appear after a short delay. This is to make sure you have time to read the page carefully before moving on. If you are reading each page in its entirety, this should not slow you down.

Also, note that you cannot go back to a page after advancing.

As a demonstration, in a few seconds the "next" button will appear below. When you're ready, click it to begin.

End of Block: Introduction

Start of Block: Consent

In order to use your observations to help future students in the transition to college, we, the [Dean of Students Office], need your permission—so the next page you will view is a consent form. Consent forms are standard practice and provide people with an overview of the activity and their rights as participants.

Page Break

CONSENT FORM
DESCRIPTION: The "What is it like to come to [SCHOOL NAME]?" activity is a study of students' experiences coming to [SCHOOL NAME]. You are invited to participate by answering questions about your perspectives about college. You will also have the chance to read about the experiences of past and current students. In agreeing to participate, you are consenting to the examination of your academic and other records from [SCHOOL NAME] in conjunction with this project. If you wish to not allow the release of these records, you may contact the researchers at any time. All information collected will be kept in strict confidentiality and will not be disclosed to anyone outside the research team.

TIME INVOLVEMENT: Your participation will take approximately 30 minutes. Please complete this activity in a single session.

RISKS AND BENEFITS: There are no risks associated with this study. We cannot and do not guarantee or promise that you will receive any benefits from this study. However, you may benefit from the knowledge that you are contributing to research on students' experiences at [SCHOOL NAME]. Your decision whether or not to participate in this study will not affect your grades or any other outcomes at [SCHOOL NAME] now or in the future.

PARTICIPANT'S RIGHTS: If you have read this form and have decided to participate, please understand your participation is voluntary and you have the right to withdraw your consent or discontinue participation at any time without penalty or loss of benefits to which you are otherwise entitled. The alternative is not to participate. You have the right to not answer

particular questions. Your individual privacy will be maintained at all times.

CONTACT INFORMATION: *Questions:* If you have any questions, concerns or complaints about this activity, its procedures, risks and benefits, contact [NAME] at [EMAIL ADDRESS].

By clicking the 'next' button you agree to participate.

Page Break

Thank you. Now let us tell you more about this activity.

Page Break

Overview
A team from the [Dean of Students Office] is interested in students' experiences in the transition to college. We have already collected data from a group of upper-year students at [SCHOOL NAME]. Now we are interested in the experiences and attitudes of incoming [SCHOOL NAME] students—you and your future classmates.

These materials have three purposes:

- To share with you the experiences and stories of upper-year students about their transition to college (in the "Current Students Survey").
- To better understand your thoughts and feelings about coming to [SCHOOL NAME].
- To help us provide incoming [SCHOOL NAME] students in future years with more accurate expectations about the transition to college.

As you can imagine, the transition to college can go more smoothly if you know what to expect. We want to learn more about students' experiences coming to [SCHOOL NAME], so we can pass this information on to future students to help them in their transition to college.

Page Break

Overview
To help us better understand students' transition to [SCHOOL NAME], we would like to ask you for your help in understanding the results and meaning of the Current Students Survey. As an incoming student who is just starting the transition to college, your thoughts and feelings about coming to college are very important to us.

So first we will share with you some of the results of the Current Students Survey. Please read the summary of results on the next pages, and take your time. Afterward, we will ask you for your thoughts and reactions to these materials.

We expect the whole activity to take about 30 minutes, but take as much time as you like. The

more thoughtful and reflective you are, the better we can understand your perspectives and the more we will be able to help future [SCHOOL NAME] students in their transition to college.

Page Break

Current Students Survey

What is the Current Students Survey? The survey focused on students' transitions to college. Who completed the Current Students Survey? These data were collected from a broad sample of sophomores, juniors, and seniors at [SCHOOL NAME] and similar colleges. The experiences described are generally representative of the experiences of [SCHOOL NAME] students as a whole.

In general, students from different backgrounds (e.g., gender, year in school, race, social class) reported many similar challenges and experiences.

Page Break

Current Students Survey

Almost all students reported a positive experience in college meeting other students, taking classes, and pursuing new opportunities.

But here we will focus on some of the challenges students face in the transition to college, and how students overcome these challenges with time.

Because there are many aspects of the college transition, we can’t share everything the current students described with you. So some incoming students will see other portions.

End of Block: Overview: Current Student Survey

Start of Block: Standard - [SCHOOL NAME]

Standard Belonging Condition

Current Students Survey: A Summary of Results
What did current students say?
Almost all students reported that they worried at first in college about whether they fit in and belonged. Students commonly reported that, when they started college, they:

- Worried about whether other students would include them and take them seriously in classes and coursework.
- Worried that other students might view their abilities negatively.
- Worried about forming study groups or finding partners to work with in labs.
- Worried about making friends.
- Felt intimidated by professors.

But with time, students came to feel that they belonged in college. They reported:

- Feeling comfortable in the academic environment.
- Feeling comfortable working with other students and interacting with professors.
- Making good friends in college.
- Feeling confident that professors and other students viewed their abilities positively.
- Feeling comfortable participating in class.

Conclusions
Most students worry at first about whether they belong in college. With time, they overcome these concerns and come to feel at home in college.

Page Break

Current Students Survey: Representative Stories

Next you will read nine stories that illustrate the major findings of the Current Students Survey. These stories are representative of the responses of participating students. Stories have been edited for clarity.

Please take your time and read these carefully.

Page Break

Current Students Survey: Representative Stories
When I got into college, I was so excited about becoming a student at such a great school. But sometimes I also worried I might be different from other students. And when I got to campus, sometimes it felt like everyone else was right at home, but I wasn't sure if I fit in. At some point, I realized that almost everyone comes to college unsure whether they fit in or not. It's ironic—everybody comes to college and feels they are different from everybody else when, really, in at

least some ways we are all pretty similar. Since I realized that, my experience at college has been almost one-hundred percent positive.

- Sophomore, African American female

Page Break

Current Students Survey: Representative Stories

I love college and I wouldn’t trade my experiences here for anything. I’ve met some close friends, I’ve had some fantastic experiences, and I’ve certainly learned a lot. Still, the transition to college can be difficult, and it was for me. My freshman year sometimes I didn’t know what I was doing—I made a lot of casual friends at parties and other places but I avoided interacting with professors in class or going to office hours. I think I was intimidated by them. I also got some low grades early on, which stressed me out. But these things all got better over time. I began to make good friends through classes. And my grades got better as I started working in study groups and asking for help from professors. I even got involved in research with a professor. Now I am happier than I have ever been at college. It is really rewarding for me to feel like I belong in the intellectual community here.

- Junior, White female

Page Break

Current Students Survey: Representative Stories

Compared to other students, I worried that my high school wasn’t so good and that maybe my high school classes hadn’t prepared me well for college. When I got to campus, to be honest sometimes I thought some of my professors were scary. I thought they were critical and hard in their grading, and I worried about how they and other students would evaluate me. So I didn’t speak much in class and I didn’t want other people to read my papers. But after a while I began feeling more comfortable—I started to enjoy my classes more, and I found some close friends I trusted. I also became more comfortable speaking in class, and sometimes I asked my friends to look over my papers for me. And I saw that even when professors are critical, or their grading harsh, it didn’t mean they looked down on me or that I didn’t belong. It was just their way of motivating high-achieving students.

- Sophomore, White male

Page Break

Current Students Survey: Representative Stories

I had small, close-knit classes in high school, so I wasn't sure what kind of relationships I would have with professors in college. I came to a liberal arts school because I wanted smaller and more personalized classes, but still I worried that I'd be just another student and just another paper to grade. And it did take time to get used to interacting with professors. But eventually I saw that the professors really did care about me and were excited to talk with me. Freshman year, I was struggling with a writing assignment, so I went to office hours to talk with the professor. I showed him a draft and he was excited about the ideas. I mean, it wasn't well written yet but he showed me how I could take it to the next level. I realized that in college

sometimes you have to reach out. Not everybody's going to be receptive all the time, but many professors are eager to work with students on subjects they love. Later, I ended up talking with another professor in my major about my interests. That ultimately led me to get involved in some research she was doing. I have just loved working with her outside the formal classroom. It is great to actually participate in cutting edge research!

- Junior, African American male

Page Break

Current Students Survey: Representative Stories

Initially my transition to college was pretty easy. Hanging out with friends in my dorm was fun, and I met a lot of people early on. After Winter Break, things got harder because it felt like all my really good friends were at home and I didn’t have friends like that at school. However, I decided to just give it time and let things fall into place. I got involved in extracurriculars, and I met people who had common interests and unique perspectives. I also got to know people in class as study partners who became close friends. I found a comfort zone by exploring my interests and taking the leap into an active life on campus. But this took time and before I found my niche here there were times when I felt quite lonely.

- Senior, Hispanic female

Page Break

Current Students Survey: Representative Stories

The summer before freshman year I was so excited about coming to college. But I was anxious too—it’s a big transition. For me the most difficult part was coming from a situation in which I knew every student in high school for the past seven years to college where I didn’t know one student yet. Once I got here, even though I met large numbers of people, I didn’t have a small group of close friends. At first sometimes I felt I had to work to find lab partners and people to be in study groups with. I was pretty homesick, and I had to remind myself that making close friends takes time. But over time, in classes, clubs, and social activities, I have met other people, some of whom are now just as close as my friends in high school were.

- Sophomore, Biracial (African American and White) male

Page Break

Current Students Survey: Representative Stories

Before coming to college, I didn’t worry much about classes and grades, but freshman year I felt unprepared for the workload and the differences in grading. It was a learning experience. After getting burned grade-wise several times and feeling stressed out in the process, I worried that I wasn’t smart enough. Fortunately, a conversation with an upperclassman set me straight—he told me that everyone struggles at first. You have to learn how to study in college. I figured out how to budget my time more wisely, so I wouldn’t have a huge crunch at the last second. And maybe even more important, I learned that when I get stuck on a problem or an idea it helps to talk with people—like a professor or someone in my class or even just a friend. Although my

start was somewhat rocky, it has felt good to learn from my mistakes, and I am proud of the success I have had.

- Senior, Asian American male

Page Break

Current Students Survey: Representative Stories

As excited as I was to come to college, I must admit that part of me thought that I might not measure up to the other students. Early on, I bombed a test. It was the worst grade I'd ever received, and I felt terrible and isolated. But then, I found out I wasn't the only one. No one did well on that test. It was really hard—the professor was trying to set a high standard. He knew it'd be tough, but he knew that if we worked hard we could get to that level. It wasn't for some time that I believed that I was totally up to par and could really succeed here. But eventually I did, and college started to feel more like home. Though I still have doubts about myself sometimes, I know they're the kinds of things everybody feels on occasion.

- Junior, White female

Page Break

Current Students Survey: Representative Stories

When I think back to the summer before freshman year, I was incredibly excited about coming to college but I was also somewhat intimidated. Walking into classes for the first time freshman year was uncomfortable, especially small classes. I worried about whether I could hold my own with other students (some of whom were upperclassmen) let alone professors. In the beginning, sometimes class discussions felt over my head. But now I feel much more relaxed. I've realized it's not about holding your own. We all bring something to the discussion, a different perspective or new ideas. It can be easy to forget what you bring. And I saw that everybody here has a common goal—to share knowledge and to learn and grow to do cool things in the future. We are all a part of that. Now I feel much more confident participating in discussions, listening, and sharing my opinions.

- Senior, White female

Page Break

Summary

Many students described worrying at first about whether they fit in and belong in college. However, with time students reported making good friends, getting to know professors, and feeling more at home in college.

Now we want to hear from you.

Page Break

Your Thoughts About Coming to [SCHOOL NAME]

We know that you are excited to come to college. But we would also like to learn more from you and your classmates about the common worries and challenges students face when they come

to college and how students overcome these challenges with time. That will help us help future students have a smoother transition to college.

In the space below please describe the thoughts, feelings, and worries you have as you prepare to come to [SCHOOL NAME]. Please describe:

- Worries you have about fitting in and belonging as you prepare to come to [SCHOOL NAME]. Why these worries are likely to be common when students first go to college.
- Why students typically feel more at home on campus with time.
- What students do to feel more at home, e.g., as they get to know friends and professors.

As you reflect and write, please consider specific experiences you will have at [SCHOOL NAME] during your first year like living in a residence hall or fraternity, meeting new people, joining student groups, interacting with professors, and taking college classes. You may also draw on your past experiences with other transitions (like starting high school or going to a summer program) and on the stories from the older students you just read, which are copied below. Please take as much time as you like.

Note: Your essay may be provided, anonymously, to incoming [SCHOOL NAME] students in future years to help give them a better understanding of what coming to college is like. The more you can describe the challenges you anticipate facing in coming to college and how you can respond to these challenges over time, the more future students will benefit. Thank you for your time and effort.

____

____

____

____

____

Representative Stories
{Quotes displayed at bottom of page for reference}

End of Block: Standard - [SCHOOL NAME]

Start of Block: Customized Condition – [SCHOOL NAME]

Example Customized Condition Student Stories

(Note: *The customized and standard conditions were identical except that student stories were individually tailored to the worries of students at each particular partner schools' campus. Examples of these customized stories are included below. Please contact authors for all customized stories)*

Page Break

Current Students Survey: Representative Stories

When I got into [SCHOOL NAME], I was so excited about becoming a student at such a great liberal arts school. But sometimes I also worried I might be different from other students. And when I got to campus, sometimes it felt like everyone else was right at home, but I wasn't sure if I fit in. At some point, I realized that almost everyone comes to college unsure whether they fit in or not. It's ironic—everybody comes to college and feels they are different from everybody else when, really, in at least some ways we are all pretty similar. Since I realized that, my experience at college has been almost one-hundred percent positive.

- *Sophomore, African American female*

Page Break

Current Students Survey: Representative Stories

I love college and I wouldn't trade my experiences here for anything. I've met some close friends, I've had some fantastic experiences, and I've certainly learned a lot. Still, the transition to college can be difficult, and it was for me. My freshman year sometimes I didn't know what I was doing—I made a lot of casual friends at parties and other places but I avoided interacting with professors in class or going to office hours. I think I was intimidated by them. I also got some low grades early on, which stressed me out. But these things all got better over time. I began to make good friends through classes. And my grades got better as I started working in study groups and asking for help from professors. I ended up talking about my interests with a professor in my major, and this ultimately led me to get involved in some research she was doing. I have just loved working with her outside the formal classroom. It's really rewarding for me to feel like I belong in the intellectual community here. Of course there are still rough days, but overall I'm happier than I've ever been at college.

- *Junior, White female*

Page Break

Current Students Survey: Representative Stories

Before coming to [SCHOOL NAME], I didn't worry much about classes and grades, but freshman year I felt unprepared for the workload and the differences in grading. It was a learning experience. After getting burned grade-wise several times and feeling stressed out in the process, I worried that I wasn't smart enough. Fortunately, a conversation with an upperclassman helped me put things in perspective—he told me that everyone struggles at first. You have to learn how to study in college. I figured out how to budget my time more wisely, so I wouldn't have a huge crunch at the last second. And maybe even more important, I learned that

when I get stuck on a problem or an idea it helps to talk with people—like a professor or someone in my class or even just a friend. Although my start was somewhat rocky, it has felt good to learn from my mistakes, and I am proud of the success I have had.
- Senior, Asian American male

End of Block: Customized Condition – [SCHOOL NAME]

Start of Block: Control - [SCHOOL NAME]

Control Condition

Current Students Survey: A Summary of Results
What did current students say?
Almost all students reported that they noticed at first in college the physical environment of [SCHOOL NAME]. Students commonly reported that, when they started at [SCHOOL NAME]:

- They "sometimes" or "frequently" thought about [SCHOOL NAME]'s campus layout and buildings.
- They "sometimes" or "frequently" thought about what living in [CITY NAME] is like.
- The weather during their first year at [SCHOOL NAME] was "somewhat" different from what they were used to.

But with time, students became familiar with the physical environment of [SCHOOL NAME]. They reported that they:

- Think less often about [SCHOOL NAME]'s campus layout and buildings.
- Think less often about living in [CITY NAME].
- Are now accustomed to [SCHOOL NAME]'s weather.

Conclusions
Most students adjust to the physical environment at [SCHOOL NAME] over time, including the campus design, location, and weather.

Page Break

Current Students Survey: Representative Stories

Next you will read nine stories that illustrate the major findings of the Current Students Survey. These stories are representative of the responses of participating students. Stories have been edited for clarity.

Please take your time and read these carefully.

Page Break

Current Students Survey: Representative Stories
I'm from a big city, so [SCHOOL NAME] was an adjustment for me. Where I'm from, there are lots of people everywhere, all the time. It is noisy most of the day (and night), and that obviously isn't true of [SCHOOL NAME]. At first, I really noticed the difference, but I've come to appreciate the opportunity to get away from noise when I want to. I think it is good for me to go to school here because it is easier to concentrate on my work when there isn't the bustle of a big city right outside my front door.

- Sophomore, African American female

Page Break

Current Students Survey: Representative Stories
I think [SCHOOL NAME] is beautiful—I love the design of the campus and the look of some of the buildings. My whole first year I think I was conscious of being in such a physically interesting place. Now that I've spent more time here I think less about the campus and buildings themselves. I guess that is natural—it isn't new to me anymore. But still, when I'm reminded of it, I am impressed again with how unique the look of [SCHOOL NAME] is.

- Junior, White female

Page Break

Current Students Survey: Representative Stories
My first year at [SCHOOL NAME] I was not prepared for how long the cold weather lasted – winter seemed to start early and end late and the wind was so cold. I had to get a warmer winter coat and better boots. But now I'm prepared for whatever the winter is like and have dealt better with the weather since then.

- Sophomore, White male

Page Break

Current Students Survey: Representative Stories
I didn't know how I would like living in [CITY NAME]. So far in my college experience it hasn't mattered as much as I thought it would have. I've been so busy at [SCHOOL NAME] that I haven't ventured off-campus that much. But when I do, I've been pleased to find some interesting places to go and fun things to do outdoors.

- Junior, African American male

Page Break

Current Students Survey: Representative Stories
I think [SCHOOL NAME]'s campus is unique. I am always impressed by the natural surroundings especially near the bridge. I love studying in the [BUILDING NAME] or even just walking on the sidewalk near historic [BUILDING NAME] on my way to or from class. Freshman year, I would sometimes go out of my way to walk to the sculptures or to [BUILDING NAME] to walk by all the different trees and plants. Now that I'm more accustomed to campus, I don't wander around as much. Whenever I have visitors, though, I make time to show them all my

favorite spots around campus.
- Senior, Asian American female

Page Break

Current Students Survey: Representative Stories
Having grown up on the West Coast, I knew that [SCHOOL NAME] would be different from where I'm from. I wasn't sure what it was going to be like to live in [STATE NAME]. Overall I've really enjoyed being at [SCHOOL NAME] and living in [CITY NAME]. Though there was a lot that was new for me – from the weather to the food to getting around campus – now I am used to it and it all seems pretty normal.
- Sophomore, Hispanic male

Page Break

Current Students Survey: Representative Stories
Having grown up in the [CITY NAME] area, I didn't think that moving to [SCHOOL NAME] would be much of a change, but it was in some ways. I'd been on campus a number of times, but I hadn't really spent a lot of time. And it's not the same visiting as it is living here. It took some getting used to at first, but now I hardly give it any thought.
- Junior, White male

Page Break

Current Students Survey: Representative Stories
Initially, [SCHOOL NAME]'s campus felt pretty confusing to me. Freshman year, it took a while to figure out where all of the buildings and offices were. Now that I've been here for a while, I know where everything is, no problem. I like that [SCHOOL NAME] is small enough that I can get almost anywhere on campus in a few minutes, but also large enough to sometimes discover new places.
- Senior, Biracial (African American and White) female

Page Break

Current Students Survey: Representative Stories
I've really liked getting to know all about [SCHOOL NAME]. I enjoy attending the [FAIR NAME] at the beginning of the semester and [GROUP NAME] dance in November, or just going to the [BUILDING NAME] for Saturday breakfast when I have the time. I especially enjoy some of the [SCHOOL NAME] traditions. [SCHOOL NAME] and this part of [STATE NAME] is a place with an interesting history and I've enjoyed learning more about it. But now that I've been here for a while, it's not something on my mind as much as it was when I first got here.
- Senior, White female

Page Break

Summary

Many students described noticing the physical environment at [SCHOOL NAME] during their freshman year—the campus design and location, the weather, and what living in [CITY NAME] is like. However, with time students became familiar with the physical environment at [SCHOOL NAME].

Now we want to hear from you.

Page Break

Your Thoughts About Coming to [SCHOOL NAME]

In an effort to further understand how this change takes place, we would like to ask you why you think this would be so—that is, why might students initially pay attention to the physical environment of [SCHOOL NAME] but ultimately become more familiar with it and pay less attention to it? As you reflect and write, please illustrate your description with examples from your own thoughts about coming to college.

You may also draw on your past experiences with other transitions (like starting going to high school or going to a summer program) and on the stories from the older students you just read, which are copied below.

Note: Your essay may be provided, anonymously, to incoming [SCHOOL NAME] students in future years to help give them a better understanding of what coming to college is like. The more you can describe what you anticipate experiencing, the more future students will benefit. Thank you for your time and effort.

____

____

____

____

____

{Quotes displayed at bottom of page for reference}

End of Block: Control - [SCHOOL NAME]

Start of Block: DV Intro Text

Thank you very much. We appreciate you taking the time to share with us your thoughts and feelings about coming to [SCHOOL NAME]. Next, we would like to ask you a few questions about the materials you just completed and your general thoughts about coming to [SCHOOL NAME].

Page Break

End of Block: DV Intro Text

Start of Block: Manipulation Check

What was the most central message from the Current Students Survey you read about?

- That students worry initially that they don't belong at [SCHOOL NAME] but come to feel at home at [SCHOOL NAME] with time. (1)
- That students get used to the physical environment (e.g., campus, location) at [SCHOOL NAME] with time. (2)
- That students come to understand social and political issues in a more sophisticated way in college. (3)
- None of the above (4)

Page Break

End of Block: Manipulation Check

Start of Block: Excited to come to [SCHOOL NAME]

How excited are you about coming to [SCHOOL NAME]?

- Not at all excited (1)
- A little bit excited (2)
- Somewhat excited (3)
- Moderately excited (4)
- Very excited (5)
- Greatly excited (6)
- Extremely excited (7)

How much do you think you'll enjoy your time at [SCHOOL NAME]?

- Not at all (1)
- A little bit (2)
- Somewhat (3)
- A moderate amount (4)
- A lot (5)
- A great deal (6)
- An extreme amount (7)

How much fun do you think you'll have at [SCHOOL NAME]?

- ◯ Not at all (1)
- ◯ A little bit (2)
- ◯ Somewhat (3)
- ◯ A moderate amount (4)
- ◯ A lot (5)
- ◯ A great deal (6)
- ◯ An extreme amount (7)

Page Break

End of Block: Excited to come to [SCHOOL NAME]

Start of Block: Fall Expected Belonging

Think ahead to when you arrive on campus this fall.

How much do you think you will feel you fit in at [SCHOOL NAME] when you arrive on campus this fall?

- ◯ Not at all (1)
- ◯ A little (2)
- ◯ Somewhat (3)
- ◯ A moderate amount (4)
- ◯ A lot (5)
- ◯ A great deal (6)
- ◯ An extreme amount (7)

How much do you think you will feel you belong at [SCHOOL NAME] when you arrive on campus this fall?

- ◯ Not at all (1)
- ◯ A little (2)
- ◯ Somewhat (3)
- ◯ A moderate amount (4)
- ◯ A lot (5)
- ◯ A great deal (6)
- ◯ An extreme amount (7)

How much do you think you will feel at home at [SCHOOL NAME] when you arrive on campus this fall?

- Not at all (1)
- A little (2)
- Somewhat (3)
- A moderate amount (4)
- A lot (5)
- A great deal (6)
- An extreme amount (7)

Page Break

End of Block: Fall Expected Belonging

Start of Block: Anticipated Difficulty of Transition

How difficult do you think that the transition to [SCHOOL NAME] could be at first?

- Not at all difficult (1)
- Slightly difficult (2)
- Moderately difficult (3)
- Very difficult (4)
- Extremely difficult (5)

How much do you think you will experience difficulties at first in the transition to [SCHOOL NAME]?

○ Not at all (1)

○ A little (2)

○ Somewhat (3)

○ A moderate amount (4)

○ A lot (5)

○ A great deal (6)

○ An extreme amount (7)

How confident are you that you will be able to handle any difficulties you face in the transition to [SCHOOL NAME]?

○ Not at all (1)

○ A little (2)

○ Somewhat (3)

○ A moderate amount (4)

○ A lot (5)

○ A great deal (6)

○ An extreme amount (7)

Page Break

End of Block: Anticipated Difficulty of Transition

Start of Block: Pos View of Difficulty

How much do you agree or disagree with this statement:

I view the difficulties of the upcoming transition to college as a positive challenge.

- Strongly agree (1)
- Agree (2)
- Mostly agree (3)
- Neither agree nor disagree (4)
- Mostly disagree (5)
- Disagree (6)
- Strongly disagree (7)

Page Break

End of Block: Pos View of Difficulty

Start of Block: Sophomore Expected Belonging

Think ahead to the end of your sophomore year at [SCHOOL NAME].

At the end of your sophomore year, how much do you think you will feel you fit in at [SCHOOL NAME]?

- Not at all (1)
- A little (2)
- Somewhat (3)
- A moderate amount (4)
- A lot (5)
- A great deal (6)
- An extreme amount (7)

At the end of your sophomore year, how much do you think you will feel you belong at [SCHOOL NAME]?

- Not at all (1)
- A little (2)
- Somewhat (3)
- A moderate amount (4)
- A lot (5)
- A great deal (6)
- An extreme amount (7)

At the end of your sophomore year, how much do you think you will feel at home at [SCHOOL NAME]?

- Not at all (1)
- A little (2)
- Somewhat (3)
- A moderate amount (4)
- A lot (5)
- A great deal (6)
- An extreme amount (7)

Page Break

End of Block: Sophomore Expected Belonging

Start of Block: Important to do Well

How important is it to you to do well at [SCHOOL NAME]?

- Not at all important (1)
- Slightly important (2)
- Somewhat important (3)
- Moderately important (4)
- Quite important (5)
- Very important (6)
- Extremely important (7)

Page Break

End of Block: Important to do Well

Start of Block: Interest in Potential Majors

How interested are you in majoring in each of the following areas:

	No interest 1 (1)	2 (2)	3 (3)	4 (4)	A great deal of interest 5 (5)
**Arts** (e.g., visual art, theater, music) (1)	○	○	○	○	○
**Humanities** (e.g., philosophy, foreign languages, English) (2)	○	○	○	○	○
**Economics** (3)	○	○	○	○	○
**Engineering** (4)	○	○	○	○	○
**Math and Science** (e.g. biology, chemistry, physics, computer science, math) (5)	○	○	○	○	○
**Social Sciences** (e.g., psychology, political science, history, sociology) (6)	○	○	○	○	○
**Health fields** (e.g., pre-med, pre-dentistry) (7)	○	○	○	○	○
**Education** (8)	○	○	○	○	○
**Business** (e.g. marketing, operations management) (9)	○	○	○	○	○
**Other** [please specify]: (10)	○	○	○	○	○

Page Break

End of Block: Interest in Potential Majors

Start of Block: Looking Forward to

Which of the following are you looking forward to about [SCHOOL NAME]? Select all that apply.

- Being independent; having more freedom (1)
- Developing relationships with other students, mentors, or professors (2)
- Learning about or developing myself personally (3)
- Being exposed to new activities, a new area, or new people (4)
- Furthering my education / taking challenging and interesting classes (5)
- Other [please specify]: (6) ____

Page Break

End of Block: Looking Forward to

Start of Block: Growth Mindset

Below are some statements about students in general. Read each statement and indicate how much you agree or disagree.
(Please notice that the question options have changed.)

You have a certain amount of intelligence, and you really can't do much to change it.

- Strongly agree (1)
- Agree (2)
- Mostly agree (3)
- Mostly disagree (4)
- Disagree (5)
- Strongly disagree (6)

You can grow your basic intelligence a lot in your lifetime.

- Strongly agree (1)
- Agree (2)
- Mostly agree (3)
- Mostly disagree (4)
- Disagree (5)
- Strongly disagree (6)

Page Break

End of Block: Growth Mindset

Start of Block: Institutional Growth Mindset

Below are some statements about your impression of people at [SCHOOL NAME]. Read each statement and indicate how much you agree or disagree.

In general, most people at [SCHOOL NAME] seem to believe that every student can learn new things and significantly grow their intelligence.

- Strongly agree (1)
- Agree (2)
- Mostly agree (3)
- Mostly disagree (4)
- Disagree (5)
- Strongly disagree (6)

So far, it seems that most people at [SCHOOL NAME] believe that students can't really change how intelligent they are.

- Strongly agree (1)
- Agree (2)
- Mostly agree (3)
- Mostly disagree (4)
- Disagree (5)
- Strongly disagree (6)

(Please note that the question options will be different on following pages)

Page Break

End of Block: Institutional Growth Mindset

Start of Block: Grit

Read each statement below and indicate how true each statement is about you.

I finish whatever I begin.

- Not at all true (1)
- Slightly true (2)
- Somewhat true (3)
- Very true (4)
- Completely true (5)

I stay interested in my goals, even if they take a long time (months or years) to complete.

- Not at all true (1)
- Slightly true (2)
- Somewhat true (3)
- Very true (4)
- Completely true (5)

I am a hard worker.

- Not at all true (1)
- Slightly true (2)
- Somewhat true (3)
- Very true (4)
- Completely true (5)

Page Break

End of Block: Grit

Start of Block: Shift and Persist

Read each statement below and indicate how true each statement is about you.

When faced with a bad situation, I do what I can do to change it for the better.

- Not at all true (1)
- Slightly true (2)
- Somewhat true (3)
- Very true (4)
- Completely true (5)

I rarely give up on something I am doing, even when things get tough.

- Not at all true (1)
- Slightly true (2)
- Somewhat true (3)
- Very true (4)
- Completely true (5)

I find I usually learn something meaningful from a difficult situation.

- Not at all true (1)
- Slightly true (2)
- Somewhat true (3)
- Very true (4)
- Completely true (5)

When I am faced with a bad situation, it helps to find a different way of looking at things.

- Not at all true (1)
- Slightly true (2)
- Somewhat true (3)
- Very true (4)
- Completely true (5)

Page Break

End of Block: Shift and Persist

Start of Block: Dialect

Compared to how most people speak and write in college, how different do you expect the way you speak and write will be?

- Not at all different (1)
- Slightly different (2)
- Moderately different (3)
- Very different (4)
- Extremely different (5)

Compared to the ways in which it is ideal to speak and write in college how different do you expect the way you speak and write will be?

- Not at all different (1)
- Slightly different (2)
- Moderately different (3)
- Very different (4)
- Extremely different (5)

How much do you expect the way you speak and write will be devalued by instructors in college?

- Not at all devalued (1)
- Slightly devalued (2)
- Moderately devalued (3)
- Very devalued (4)
- Extremely devalued (5)

How much do you expect the way you speak and write will be devalued by your classmates in college?

- Not at all devalued (1)
- Slightly devalued (2)
- Moderately devalued (3)
- Very devalued (4)
- Extremely devalued (5)

Page Break

End of Block: Dialect

Start of Block: Bureaucratic Frustration

Think about your experiences with [SCHOOL NAME] so far. In general, how simple or complicated has it been for you to…

	Extremely simple (1)	Very simple (2)	Kind of simple (3)	Neither simple nor complicated (4)	Kind of complicated (5)	Very complicated (6)	Extremely complicated (7)
Figure out which courses you need for your degree or your future career goals (1)	○	○	○	○	○	○	○
Get accurate information about courses or financial aid from the counselors and advisors at [SCHOOL NAME] (2)	○	○	○	○	○	○	○
Actually receive the financial aid and scholarships that you were eligible for at [SCHOOL NAME] (3)	○	○	○	○	○	○	○
Go through the application and enrollment process at [SCHOOL NAME] (4)	○	○	○	○	○	○	○

Page Break

Think about your overall experience of getting what you need from the [SCHOOL NAME] offices and administration, including filling out paperwork, getting housing, getting information about your classes or degree plans, or getting financial aid.
In general, how frustrating has your experience been with the [SCHOOL NAME] offices and administration?

- Not at all frustrating (1)
- Slightly frustrating (2)
- Somewhat frustrating (3)
- Very frustrating (4)
- Extremely frustrating (5)

Page Break

End of Block: Bureaucratic Frustration

Start of Block: Personality

How well do the following statements describe your personality? *(There are no right or wrong answers; we just want to know how you think about yourself.)*

I see myself as someone who...

	Extremely well (1)	Very well (2)	Moderately well (3)	Slightly well (4)	Not well at all (5)
...is reserved (1)	o	o	o	o	o
...is generally trusting (2)	o	o	o	o	o
...tends to be lazy (3)	o	o	o	o	o
...is relaxed, handles stress well (4)	o	o	o	o	o
...has few artistic interests (5)	o	o	o	o	o
...is outgoing, sociable (6)	o	o	o	o	o
...tends to find fault with others (7)	o	o	o	o	o
...does a thorough job (8)	o	o	o	o	o
...gets nervous easily (9)	o	o	o	o	o
...has an active imagination (10)	o	o	o	o	o

Page Break

End of Block: Personality

Start of Block: Student Identity Centrality

Read each statement below and indicate how much you agree or disagree.

Overall, being a student has very little to do with how I feel about myself.

- Strongly disagree (1)
- Disagree (2)
- Somewhat disagree (3)
- Neither agree nor disagree (4)
- Somewhat agree (5)
- Agree (6)
- Strongly agree (7)

Being a student is an important reflection of who I am.

- Strongly disagree (1)
- Disagree (2)
- Somewhat disagree (3)
- Neither agree nor disagree (4)
- Somewhat agree (5)
- Agree (6)
- Strongly agree (7)

Being a student is unimportant to my sense of what kind of a person I am.

- Strongly disagree (1)
- Disagree (2)
- Somewhat disagree (3)
- Neither agree nor disagree (4)
- Somewhat agree (5)
- Agree (6)
- Strongly agree (7)

In general, being a student is an important part of my self-image.

- Strongly disagree (1)
- Disagree (2)
- Somewhat disagree (3)
- Neither agree nor disagree (4)
- Somewhat agree (5)
- Agree (6)
- Strongly agree (7)

End of Block: Student Identity Centrality

Start of Block: Learn Anything (All)

Did you learn anything in reading the "What is it like to come to [SCHOOL NAME]?" materials?

- Yes, I learned something. (1)
- No, I did not learn anything. (2)

Display This Question:

If __pdd__t1__dv__learn_anything__pdd__ Did you learn anything in reading the "What is it like to co... = Yes, I learned something.

Please describe what you learned from reading these materials in a few sentences.

____

____

____

____

____

Page Break

End of Block: Learn Anything (All)

Start of Block: Demographic Intro Text

Now we want to ask some questions about you, just so we have a better picture of the incoming class.

Your responses are confidential, and you are free to decline to answer any question that you don't wish to answer.

End of Block: Demographic Intro Text

Start of Block: Gender / YOB / US Citizen / Zipcode

With which gender do you identify?

- Man (1)
- Transman (5)
- Transwoman (6)
- Woman (2)
- I prefer another term [if you wish, please specify]: (4) ____

What gender were you assigned at birth?

- Male (1)
- Female (2)
- Other (3) ____

In what year were you born? (YYYY)

____

Which best describes you?

○ I am a US citizen. (1)

○ I am a permanent resident of the US (green card holder) (2)

○ Visa holder (3)

○ Other [please specify]: (4) ____

What is the zipcode of your hometown (US only)?

____

Page Break

End of Block: Gender / YOB / US Citizen / Zipcode

Start of Block: Race

With which racial/ethnic group(s) do you identify? Please check the box(es) that apply.

- ▢ American Indian or Alaskan Native (1)
- ▢ East Asian (e.g., Chinese, Japanese, Korean, Taiwanese) (2)
- ▢ Southeast Asian (e.g., Filipino, Vietnamese, Indonesian) (3)
- ▢ South Asian (e.g., Pakistani, Indian, Nepalese, Sri Lankan) (4)
- ▢ Other Asian (5)
- ▢ Mexican American/Chicano (6)
- ▢ Puerto Rican (7)
- ▢ Central American (8)
- ▢ Other Hispanic or Latino (9)
- ▢ African American/Black (10)
- ▢ African (11)
- ▢ Caribbean (12)
- ▢ Other Black (13)
- ▢ European/European American (14)
- ▢ Middle Eastern/Middle Eastern American (15)
- ▢ Other White (16)
- ▢ Native Hawaiian or Other Pacific Islander (17)
- ▢ [please specify]: (18) ____

Page Break

Please indicate the race/ethnicity with which you identify most strongly, if any.

- American Indian or Alaskan Native (1)
- East Asian (e.g., Chinese, Japanese, Korean, Taiwanese) (2)
- Southeast Asian (e.g., Filipino, Vietnamese, Indonesian) (3)
- South Asian (e.g., Pakistani, Indian, Nepalese, Sri Lankan) (4)
- Other Asian (5)
- Mexican American/Chicano (6)
- Puerto Rican (7)
- Central American (8)
- Other Hispanic or Latino (9)
- African American/Black (10)
- African (11)
- Caribbean (12)
- Other Black (13)
- European/European American (14)
- Middle Eastern/Middle Eastern American (15)
- Other White (16)
- Native Hawaiian or Other Pacific Islander (17)
- Other: $q://QID62/ChoiceTextEntryValue/18} (18)
- Multiracial (19)

Page Break

End of Block: Race

Start of Block: Religion

With which religious group do you most closely identify?

○ Agnostic (not sure if there is a God) (1)

○ Atheist (do not believe in God) (2)

○ Baha'i (3)

○ Buddhist (4)

○ Christian - Catholic (5)

○ Christian - Evangelical (6)

○ Christian - Jehovah's Witness (7)

○ Christian - Mormon (Church of Jesus Christ of Latter-day Saints/LDS) (8)

○ Christian - Orthodox (e.g., Eastern Orthodox, Greek Orthodox, Russian Orthodox, or some other Orthodox church) (9)

○ Christian- Protestant (e.g., Methodist, Pentecostal, Baptist, Presbyterian, Non-denominational, Anglican, Lutheran) (10)

○ Hindu (11)

○ Jewish (12)

○ Muslim (13)

○ Native American or indigenous religion (14)

○ Sikh (15)

○ Taoist (16)

○ Unitarian (Universalist) (17)

○ Wiccan (18)

○ Spiritual but not religious (19)

○ Not spiritual or religious (20)

○ Other [please specify]: (21) ____

Page Break

Display This Question:

If __pdd__t1__demog__religion__pdd__ With which religious group do you most closely identify? != Not spiritual or religious

How closely do you identify with being a member of your religious or non-religious group?

- ○ Do not identify (1)
- ○ Slightly identify (2)
- ○ Somewhat identify (3)
- ○ Strongly identify (4)
- ○ Very strongly identify (5)

Page Break

End of Block: Religion

Start of Block: Sexual Orientation

How would you describe your sexual orientation?

◯ Heterosexual / Straight (1)

◯ Gay / Lesbian (2)

◯ Bisexual (3)

◯ Pansexual (4)

◯ Asexual (5)

◯ I prefer another term [if you wish, please specify]: (6)
____

◯ I'd rather not say (7)

◯ I don't know (8)

Page Break

End of Block: Sexual Orientation

Start of Block: Disability

Disability is defined here as having a physical or mental impairment or medical condition that substantially limits a major life activity, or having a history or record of such an impairment or medical condition.

Please check the box(es) below that apply.

- ☐ Yes, I have a physical disability (e.g., mobility impairment, use of wheelchair or other assistive device) (1)
- ☐ Yes, I have a sensory disability (e.g., blindness, deafness) (2)
- ☐ Yes, I have had an experience of a mental illness (e.g., major depression, bipolar disorder) (3)
- ☐ Yes, I have a learning disability (e.g., dyslexia, discalculia) (4)
- ☐ Yes, I have a neurodevelopmental disability (e.g. ADD/ADHD) (5)
- ☐ Yes, I have a medical condition that substantially limits a major life activity (e.g., diabetes, epilepsy, HIV/AIDS) (6)
- ☐ ⊗ No, I don't have a disability (7)
- ☐ ⊗ I'd rather not say (8)

Page Break

End of Block: Disability

Start of Block: Social Class

How would you describe your family's social class?

- ○ Working class (1)
- ○ Lower middle class (2)
- ○ Middle class (3)
- ○ Upper middle class (4)
- ○ Upper class (5)

How do you think the high school you attended compares to the high schools attended by most other incoming [SCHOOL NAME] students?

- ○ My high school is less advantaged than the high schools attended by most other incoming students (1)
- ○ My high school is neither less advantaged nor more advantaged than the high schools attended by most other incoming students (2)
- ○ My high school is more advantaged than the high schools attended by most other incoming students (3)

Display This Question:

If __pdd__t1__demog__hs_adv__pdd__ How do you think the high school you attended compares to the hig... = My high school is <u>less advantaged</u> than the high schools attended by most other incoming students</u></u>

And hs_advantage = true

Or __pdd__t1__demog__hs_adv__pdd__ How do you think the high school you attended compares to the hig... = My high school is <u>more advantaged</u> than the high schools attended by most other incoming students

And hs_advantage = true

If you indicated that your high school is relatively less or more advantaged, please describe briefly why you think so.

____

____

____

____

____

Page Break

End of Block: Social Class / HS Advantaged (Closed) / HS Advantaged (Open)

Start of Block: Parent Education

With which gender does your primary parent or guardian/caregiver identify?

- Man (1)
- Transman (5)
- Transwoman (6)
- Woman (2)
- Prefers another term [if you wish, please specify]: (4) ____

Does your primary parent or guardian/caregiver identify as transgender?

- Yes (1)
- No (2)

Page Break

What is the highest level of education your primary parent or guardian/caregiver has attained?

○ Less than high school graduate (1)

○ High school graduate (2)

○ Some college/vocational school (3)

○ Associate's (2-year college) degree (4)

○ Bachelor's (4-year college) degree (5)

○ Some graduate school (6)

○ Master's degree (7)

○ Law degree (8)

○ Medical degree (9)

○ Doctoral degree (10)

○ Don't know (11)

○ Doesn't apply (12)

Page Break

Do you have a second parent or guardian/caregiver?

○ Yes (1)

○ No (2)

Page Break

Display This Question:

If __pdd__t1__demog__ed_level_present_p2__pdd__ Do you have a second parent or guardian/caregiver? = Yes

With which gender does your second parent or guardian/caregiver identify?

- ○ Man (1)
- ○ Transman (5)
- ○ Transwoman (6)
- ○ Woman (2)
- ○ Prefers another term [if you wish, please specify]: (4) ____

Display This Question:

If __pdd__t1__demog__ed_level_present_p2__pdd__ Do you have a second parent or guardian/caregiver? = Yes

Does your second parent or guardian/caregiver identify as transgender?

- ○ Yes (1)
- ○ No (2)

Page Break

Display This Question:

If __pdd__t1__demog__ed_level_present_p2__pdd__ Do you have a second parent or guardian/caregiver? = Yes

What is the highest level of education your second parent or guardian/caregiver has attained?

- Less than high school graduate (1)
- High school graduate (2)
- Some college/vocational school (3)
- Associate's (2-year college) degree (4)
- Bachelor's (4-year college) degree (5)
- Some graduate school (6)
- Master's degree (7)
- Law degree (8)
- Medical degree (9)
- Doctoral degree (10)
- Don't know (11)
- Doesn't apply (12)

Page Break

End of Block: Parent Education

Start of Block: English First Language

Is English your first language?

- Yes (1)
- No (2)

Page Break

Display This Question:

If __pdd__t1__demog__english_first_lang__pdd__ Is English your first language? = No

At what age did you first learn English?

▼ 1 year old (1) ... 18 years old (18)

Page Break

End of Block: English First Language

Start of Block: HS Rank / HS Graduation Date

What was your high school GPA class rank?
Please choose the range that indicates where your GPA was compared to the GPAs of other students in your graduating class. For example, marking "top 1%" means that you had one of the highest GPAs in your class. Marking "41%-50%" means you had a GPA that was a little above or about average for your class. *If you aren't sure, please provide your best guess.*

▼ top 1% (1) ... 91-100% (12)

When was, or will be, your high school graduation date? (MM/DD/YYYY)
If you aren't sure, please provide your best guess.

____

Display This Question:

If transfer_[SCHOOL NAME] = true

Are you a transfer student?

○ Yes, I have gone to college somewhere else before [SCHOOL NAME]. (1)

○ No, this fall at [SCHOOL NAME] will be my first time attending college. (2)

Page Break

End of Block: HS Rank / HS Graduation Date / Transfer

Start of Block: Friends Who Attend / Days on Campus

How many friends do you have who will attend [SCHOOL NAME] this fall?

○ None (1)

○ 1 or 2 (2)

○ 3 to 5 (3)

○ 6 to 8 (4)

○ More than 8 (5)

In the past year, how many days have you spent on [SCHOOL NAME]'s campus?

____

If you have spent time on campus already, what was it for? *Please check the box(es) below that apply.*

▢ Academic program (1)

▢ Admissions event (2)

▢ Athletic program (3)

▢ Social program (4)

▢ Tour (5)

▢ Orientation (6)

▢ Other [please specify]: (7) ____

Page Break

End of Block: Friends Who Attend / Days on Campus

Start of Block: Siblings in College

If you have siblings, are you the first among your siblings to attend college?

- Yes, I am the first of my siblings to attend college. (1)
- No, I have at least one sibling who has attended college. (2)
- I do not have siblings. (3)
- Other [please specify]: (4) ____

Display This Question:

If __pdd__t1__demog__sibling_college__pdd__ If you have siblings, are you the first among your sibli... = No, I have at least one sibling who has attended college.

How many of your siblings have attended college?

____

Page Break

End of Block: Siblings in College

Start of Block: Parent or Guardian / Job

Display This Question:

If parent_child = true

Are you a parent or guardian of a child 18 years or younger?

- Yes (1)
- No (2)

Display This Question:

If job = true

When school starts do you expect to be working in a paid position (*e.g., on- or off-campus, including work-study*)?

- Yes, a full-time job (30 hours a week or more) (1)
- Yes, a part-time job (Less than 30 hours a week) (2)
- No (3)

Page Break

End of Block: Parent or Guardian / Job

Start of Block: Height / Weight

What is your height in feet and inches? Round to the nearest half of an inch.

▼ 7 feet or above (84) ... 4 feet 10 inches or below (58)

What is your current weight in pounds?

▼ 500 lbs or above (500) ... 90 lbs or below (90)

Page Break

End of Block: Height / Weight

Start of Block: General Health

In general, would you say your physical health is:

- Poor (1)
- Fair (2)
- Good (3)
- Very good (4)
- Excellent (5)

In general, would you say your mental health is:

- Poor (1)
- Fair (2)
- Good (3)
- Very good (4)
- Excellent (5)

Page Break

End of Block: General Health

Start of Block: Freshman Residential College/ Living Freshman Year

Display This Question:

If residential_[SCHOOL NAME] = true

Which residential college will you be a part of in the fall?

○ [COLLEGE NAME] (1)

○ [COLLEGE NAME] (2)

○ [COLLEGE NAME] (3)

○ [COLLEGE NAME] (4)

○ [COLLEGE NAME] (5)

○ [COLLEGE NAME] (6)

○ [COLLEGE NAME] (7)

○ [COLLEGE NAME] (8)

○ [COLLEGE NAME] (9)

○ [COLLEGE NAME] (10)

Display This Question:

If live_freshman = true

And transfer_student != true

Where do you plan to live during your freshman year?

○ Campus residence hall (1)

○ Fraternity or sorority house (2)

○ Other college/university housing (3)

○ Parent/guardian's home (4)

○ Other off-campus housing (5)

○ Other [please specify]: (6) ____

Page Break

End of Block: Freshman Residential College / Living Freshman Year

---

Start of Block: Commute

Display This Question:

If __pdd__t1__demog__campus_living__pdd__ Where do you plan to live during your freshman year? , Parent/guardian's home Is Not Displayed

Or __pdd__t1__demog__campus_living__pdd__ Where do you plan to live during your freshman year? = Parent/guardian's home

Or __pdd__t1__demog__campus_living__pdd__ Where do you plan to live during your freshman year? = Other off-campus housing

Or __pdd__t1__demog__campus_living__pdd__ Where do you plan to live during your freshman year? = Other [please specify]:

If you plan to live off campus, how do you plan to commute to campus on most days?

◯ Walk (1)

◯ Bike (2)

◯ Drive myself (3)

◯ Get a ride (e.g. carpool, be dropped off) (4)

◯ Take public transportation (e.g., bus, train) (5)

◯ Other [please specify]: (6) ____

Display This Question:

If __pdd__t1__demog__commute__pdd__ If you plan to live off campus, how do you plan to commute to ca... , Walk Is Displayed

How many minutes do you expect your commute to take on most days?

____

Page Break

End of Block: Commute

Start of Block: Athlete

Were you recruited to play a sport at [SCHOOL NAME]?

◯ Yes (1)

◯ No (2)

Display This Question:

If __pdd__t1__demog__athlete__pdd__ Were you recruited to play a sport at ${e://Field/school_name}? = Yes

What is your sport?

____

Page Break

End of Block: Athlete

Start of Block: Ask Advice

How many people do you know who you can ask for advice about college?

____

Page Break

End of Block: Ask Advice

Start of Block: How Distracted / Technical Difficulties

Please answer some final questions about your experience completing these materials.

How distracted were you as you completed the materials? *(e.g., by interruptions, other people, social media, etc.)*

- Extremely distracted (1)
- Very distracted (2)
- Somewhat distracted (3)
- Slightly distracted (4)
- Not distracted at all (5)

Did you have any technical difficulties as you completed the materials? For instance, did you have to restart the materials, did your computer freeze up, did the internet stop working, or did anything else happen that interfered with your ability to complete them?

- Yes, I had some technical difficulties with the activity. *Please explain what happened.* (1) ____
- No, everything worked fine. (2)

End of Block: How Distracted / Technical Difficulties / Survey Access

CTC Belonging Follow-Up Survey

Start of Block: Intro

Page Break

Welcome!

[SCHOOL NAME] is part of a collaboration of colleges and researchers trying to better understand and improve students' experiences in the transition to college. Last summer, we asked you, the Class of 2022, to share your perspectives on the transition to college with us.

Now we would like to ask you to look back on your first year at [SCHOOL NAME] and tell us more about your experiences here. Along with the perspectives you and your classmates provided last summer, your responses will help us make this transition smoother for future generations of [SCHOOL NAME] students.

[**In thanks for completing the survey, you will receive a $5 Amazon gift card.**]

Page Break

Important Note
In order to get your thoughtful reflections, we ask that you complete this survey on your own, without interruption, in a quiet location. The survey will take about 15 minutes. Please complete it in one session. We suggest taking the survey on a computer, rather than a mobile device or tablet.

Page Break

End of Block: Intro

Start of Block: Universal Consent

Consent Form

DESCRIPTION: The purpose of the Social and Academic Life at [SCHOOL NAME] activity is to understand students’ experiences while at [SCHOOL NAME]. You are invited to participate by answering questions about your perspectives about college and your time at [SCHOOL NAME] so far. Your participation will take approximately 15 minutes. Please complete this activity in a single session. You will receive a $5 Amazon gift card as a thank you for completing the survey.

PARTICIPANT'S RIGHTS: Your participation is voluntary and you have the right to withdraw your consent or discontinue participation at any time without penalty or loss of benefits to which you are otherwise entitled. You may choose not to answer specific questions. Your individual privacy will be maintained at all times, and your responses will remain confidential. Your responses will not be disclosed to anyone outside the research team.

RISKS AND BENEFITS: By clicking on the next button, you are consenting to participate in this survey and to the examination of your academic and other records from [SCHOOL NAME] in conjunction with this project. Please keep in mind that any identifying information will be replaced with confidential codes prior to this examination. If you wish to not allow the release of these records, you may contact the researchers at any time.

CONTACT INFORMATION: If you have any questions or, concerns or complaints about this activity, its procedures, risks and benefits, contact [NAME] at [EMAIL ADDRESS].

By clicking the next button you 'agree' to participate.

End of Block: Universal Consent

Start of Block: Intro: Belonging/general

The first questions are about what [SCHOOL NAME] has been like for you so far. For each question or statement, mark the response that is true for you. Please use the whole range of each scale.

End of Block: Intro: Belonging/general

Start of Block: Belonging - key

I feel like I belong at [SCHOOL NAME].

- Strongly disagree (1)
- Disagree (2)
- Somewhat disagree (3)
- Somewhat agree (4)
- Agree (5)
- Strongly agree (6)

I fit in well at [SCHOOL NAME].

- Strongly disagree (1)
- Disagree (2)
- Somewhat disagree (3)
- Somewhat agree (4)
- Agree (5)
- Strongly agree (6)

I feel like an outsider at [SCHOOL NAME].

○ Strongly disagree (1)

○ Disagree (2)

○ Somewhat disagree (3)

○ Somewhat agree (4)

○ Agree (5)

○ Strongly agree (6)

I feel comfortable at [SCHOOL NAME].

○ Strongly disagree (1)

○ Disagree (2)

○ Somewhat disagree (3)

○ Somewhat agree (4)

○ Agree (5)

○ Strongly agree (6)

Page Break

End of Block: Belonging - key

Start of Block: Belonging Uncertainty - key

When you think about [SCHOOL NAME], how often, if ever, do you wonder: "Maybe I don't belong here?"

- Always (1)
- Frequently (2)
- Sometimes (3)
- Hardly ever (4)
- Never (5)

End of Block: Belonging Uncertainty - key

Start of Block: Academic Identification - key

How important is it to you that you do well at [SCHOOL NAME]?

- Not at all important (1)
- Slightly important (2)
- Somewhat important (3)
- Moderately important (4)
- Quite important (5)
- Very Important (6)
- Extremely important (7)

End of Block: Academic Identification - key

Start of Block: Student Identity Centrality (student_centrality)

Overall, being a student has very little to do with how I feel about myself.

- Strongly disagree (1)
- Disagree (2)
- Somewhat disagree (3)
- Neither agree nor disagree (4)
- Somewhat agree (5)
- Agree (6)
- Strongly agree (7)

Being a student is an important reflection of who I am.

- Strongly disagree (1)
- Disagree (2)
- Somewhat disagree (3)
- Neither agree nor disagree (4)
- Somewhat agree (5)
- Agree (6)
- Strongly agree (7)

Being a student is unimportant to my sense of what kind of a person I am.

- Strongly disagree (1)
- Disagree (2)
- Somewhat disagree (3)
- Neither agree nor disagree (4)
- Somewhat agree (5)
- Agree (6)
- Strongly agree (7)

In general, being a student is an important part of my self-image.

- Strongly disagree (1)
- Disagree (2)
- Somewhat disagree (3)
- Neither agree nor disagree (4)
- Somewhat agree (5)
- Agree (6)
- Strongly agree (7)

End of Block: Student Identity Centrality (student_centrality)

Start of Block: Self Efficacy/Ability - key

Right now, how confident do you feel that you have the ability to do well at [SCHOOL NAME]?

- ○ Not at all confident (1)
- ○ Slightly confident (2)
- ○ Somewhat confident (3)
- ○ Moderately confident (4)
- ○ Quite confident (5)
- ○ Very confident (6)
- ○ Extremely confident (7)

End of Block: Self Efficacy/Ability - key

Start of Block: Academic Potential - key

Using a percentile rank, assess your potential right now, compared with other first-year students at [SCHOOL NAME], to succeed at [SCHOOL NAME].

Marking 50% means you believe you have more potential than half of first-year students at [SCHOOL NAME], and less potential than half. Marking 90% means you believe you have more

potential than almost all first-year students at [SCHOOL NAME]. Marking 10% means you believe you have less potential than almost all first-year students at [SCHOOL NAME].

- 0% (1)
- 10% (2)
- 20% (3)
- 30% (4)
- 40% (5)
- 50% (6)
- 60% (7)
- 70% (8)
- 80% (9)
- 90% (10)
- 100% (11)

End of Block: Academic Potential - key

Start of Block: Academic Behaviors - key

In the past month of school, how often have you...

	Never (1)	Once (2)	2-3 times (3)	4-6 times (4)	7 or more times (5)
Met with a professor outside of class? (sucbehv_1)	○	○	○	○	○
Met with an academic advisor(s)? (sucbehv_2)	○	○	○	○	○
Sought academic tutoring? (e.g, at a writing center, math and science center, another center) (sucbehv_3)	○	○	○	○	○
Participated in a formal or informal study group? (sucbehv_4)	○	○	○	○	○

End of Block: Academic Behaviors - key

Start of Block: Loneliness - key

Page Break

At [SCHOOL NAME], how often do you feel isolated from others?

○ Hardly ever (1)

○ Some of the time (2)

○ Often (3)

At [SCHOOL NAME], how often do you feel that you lack companionship?

○ Hardly ever (1)

○ Some of the time (2)

○ Often (3)

End of Block: Loneliness - key

Start of Block: Intro: Friends

Thank you. The next questions ask about the activities you participate in and the relationships you have with friends and mentors at [SCHOOL NAME].

End of Block: Intro: Friends

Start of Block: Close Friends - key

Thinking back on this past academic year so far, I feel that I have made some close friends at [SCHOOL NAME].

- Strongly disagree (1)
- Disagree (2)
- Somewhat disagree (3)
- Somewhat agree (4)
- Agree (5)
- Strongly agree (6)

I feel that there is <u>no one</u> at [SCHOOL NAME] I can share my personal worries and fears with.

- Strongly disagree (1)
- Disagree (2)
- Somewhat disagree (3)
- Somewhat agree (4)
- Agree (5)
- Strongly agree (6)

End of Block: Close Friends - key

Start of Block: Friends Network - key

We would like to know about your closest friends at [SCHOOL NAME]. We have provided space for up to 7 friends below but many students do not use all of these spaces. Please just list the close friends you have at [SCHOOL NAME].

	**How close of a friend is this person?**							**Friend's Initials**
	Not very close (1)	(2)	(3)	(4)	(5)	(6)	Extremely close (7)	
Friend #1 (friends_1)	○	○	○	○	○	○	○	
…	○	○	○	○	○	○	○	
Friend #7 (friends_7)	○	○	○	○	○	○	○	

Page Break

We are interested in the different kinds of friendship networks students have. For each friend, please indicate their gender and racial/ethnic background.

	Gender	Racial/ethnic background

		Asian or Asian American (1)	Black, African, or African American (2)	Hispanic or Latino (3)	Native American or American Indian (4)	White, Caucasian, or European American (5)	Native Hawaiian or other Pacific Islander (6)	Identifies in another way (7)
[Friend 1 Initials]	▼ Man, Woman,…	▢	▢	▢	▢	▢	▢	▢
…	▼ Man, Woman,…	▢	▢	▢	▢	▢	▢	▢
[Friend 7 Initials]	▼ Man, Woman,…	▢	▢	▢	▢	▢	▢	▢

End of Block: Friends Network - key

Start of Block: Mentorship - key

Thinking back on this past academic year, have you developed a relationship with a mentor at [SCHOOL NAME] (formal or informal) that has been helpful to you and your academic and/or personal development?
(e.g., faculty mentor, staff person, an upper-year student, etc.)

○ Yes (0)

○ No (1)

Page Break

Display This Question:

If __pdd__t2__dv__mentorship1_mentor__pdd__ Thinking back on this past academic year, have you devel... = Yes

If YES, who is that? Check all that apply.

- ▢ A faculty member (1)
- ▢ Another undergraduate student (including residential staff) (2)
- ▢ An administrator (3)
- ▢ A [SCHOOL NAME] staff member (4)
- ▢ An athletic coach (5)
- ▢ An alumnus (6)
- ▢ Other (8) ____

Page Break

Think about the most important mentorship you have had during this academic year. How important has this mentorship been to you?

- ○ Not at all important (1)
- ○ Slightly important (2)
- ○ Somewhat important (3)
- ○ Moderately important (4)
- ○ Quite important (5)
- ○ Very important (6)
- ○ Extremely important (7)

End of Block: Mentorship - key

Start of Block: Extracurricular Activities - key

Please list up to three groups or extracurricular organizations you have joined or participated in so far at [SCHOOL NAME].

You can think about groups related to athletics/recreation, careers, community service, ethnicity/culture, media/arts, political or social awareness, religion/philosophy, research, or other areas.

	Group or organization name	Activity Type
	(1)	
#1 (orginv_1)		▼ Academic or professional (1) ... Social (10)
#2 (orginv_2)		▼ Academic or professional (1) ... Social (10)
#3 (orginv_3)		▼ Academic or professional (1) ... Social (10)

Page Break

For each of the groups or organizations you listed on the previous page, how involved have you been with this group?

	Not very involved (1)	Somewhat involved (2)	Moderately involved (3)	Quite involved (4)	Very involved (5)
[Group Name #1]	○	○	○	○	○
[Group Name #2]	○	○	○	○	○
[Group Name #3]	○	○	○	○	○

End of Block: Extracurricular Activities - key

Start of Block: Growth Mindset (growth mindset)

Below are some statements about students in general. Read each statement and indicate how much you agree or disagree.

You have a certain amount of intelligence, and you really can't do much to change it.

- Strongly agree (1)
- Agree (2)
- Mostly agree (3)
- Mostly disagree (4)
- Disagree (5)
- Strongly disagree (6)

You can grow your basic intelligence a lot in your lifetime.

- Strongly agree (1)
- Agree (2)
- Mostly agree (3)
- Mostly disagree (4)
- Disagree (5)
- Strongly disagree (6)

End of Block: Growth Mindset (growth mindset)

Start of Block: Internalized Growth Mindset (internal_mindset)

Read each statement below and indicate how true each statement is about you.

I am always finding something new to learn.

◯ Not at all true (1)

◯ A little bit true (2)

◯ Somewhat true (3)

◯ Pretty true (4)

◯ Very true (5)

◯ Extremely true (6)

No matter what I do, I am always learning.

◯ Not at all true (1)

◯ A little bit true (2)

◯ Somewhat true (3)

◯ Pretty true (4)

◯ Very true (5)

◯ Extremely true (6)

I learn a lot even when I am not in class.

◯ Not at all true (1)

◯ A little bit true (2)

◯ Somewhat true (3)

◯ Pretty true (4)

◯ Very true (5)

◯ Extremely true (6)

End of Block: Internalized Growth Mindset (internal_mindset)

Start of Block: Grit (grit)

Read each statement below and indicate how true each statement is about you.

I finish whatever I begin.

◯ Not at all true (1)

◯ Slightly true (2)

◯ Somewhat true (3)

◯ Very true (4)

◯ Completely true (5)

I stay interested in my goals, even if they take a long time (months or years) to complete.

◯ Not at all true (1)

◯ Slightly true (2)

◯ Somewhat true (3)

◯ Very true (4)

◯ Completely true (5)

I am a hard worker.

◯ Not at all true (1)

◯ Slightly true (2)

◯ Somewhat true (3)

◯ Very true (4)

◯ Completely true (5)

End of Block: Grit (grit)

Start of Block: Personality (personality)

How well do the following statements describe your personality? *There are no right or wrong answers; we just want to know how you think about yourself.*
I see myself as someone who...

	Disagree strongly (1)	Disagree a little (2)	Neither agree nor disagree (3)	Agree a little (4)	Agree strongly (5)
...is reserved.	◯	◯	◯	◯	◯
...is generally trusting.	◯	◯	◯	◯	◯
...tends to be lazy.	◯	◯	◯	◯	◯
...is relaxed, handles stress well.	◯	◯	◯	◯	◯
...has few artistic interests.	◯	◯	◯	◯	◯
...is outgoing, sociable.	◯	◯	◯	◯	◯
...tends to find fault with others.	◯	◯	◯	◯	◯
...does a thorough job.	◯	◯	◯	◯	◯
...gets nervous easily.	◯	◯	◯	◯	◯
...has an active imagination.	◯	◯	◯	◯	◯

End of Block: Personality (personality)

Start of Block: "Know How" mindset items (know_how)

College can be complicated. There are many tasks to complete (e.g. completing paperwork, getting financial aid, figuring out how to get what you need from the administration, learning how college classes work). We're interested in your ideas about navigating college. There are no

right or wrong answers. We just want to know how you feel about "doing college." Read each statement and indicate how much you agree or disagree.

You either know how to navigate college or not, and there isn't much you can do to change it.

- Strongly agree (1)
- Agree (2)
- Somewhat agree (3)
- Somewhat disagree (4)
- Disagree (5)
- Strongly disagree (6)

If you can't figure out how to navigate college, you probably can't get much better at it.

- Strongly agree (1)
- Agree (2)
- Somewhat agree (3)
- Somewhat disagree (4)
- Disagree (5)
- Strongly disagree (6)

You can learn new facts, but you can't really change your basic skills for navigating college.

- Strongly agree (1)
- Agree (2)
- Somewhat agree (3)
- Somewhat disagree (4)
- Disagree (5)
- Strongly disagree (6)

End of Block: "Know How" mindset items (know_how)

Start of Block: Insider knowledge (inside_know)

Read each statement below and indicate how true each statement is about you.

I often find I know more about how to do well in college than other students I talk to.

- Not at all true (1)
- Slightly true (2)
- Moderately true (3)
- Very true (4)
- Extremely true (5)

At times I feel lost about how to get things done in college.

- Not at all true (1)
- Slightly true (2)
- Moderately true (3)
- Very true (4)
- Extremely true (5)

I know how to get everything I need in college.

- Not at all true (1)
- Slightly true (2)
- Moderately true (3)
- Very true (4)
- Extremely true (5)

Other students know more than I do about how to succeed in college.

- Not at all true (1)
- Slightly true (2)
- Moderately true (3)
- Very true (4)
- Extremely true (5)

I feel like I don't know much about how college works.

- Not at all true (1)
- Slightly true (2)
- Moderately true (3)
- Very true (4)
- Extremely true (5)

End of Block: Insider knowledge (inside_know)

Start of Block: Family Achievement Guilt - key

The next questions are about your experiences in college and your family.

Page Break

Sometimes my family can't relate to my experience in college.

- Strongly disagree (1)
- Disagree (2)
- Somewhat disagree (3)
- Somewhat agree (4)
- Agree (5)
- Strongly agree (6)

Sometimes my experiences at college make me feel like I can't relate to my family.

- Strongly disagree (1)
- Disagree (2)
- Somewhat disagree (3)
- Somewhat agree (4)
- Agree (5)
- Strongly agree (6)

End of Block: Family Achievement Guilt - key

Start of Block: Family Achievement Guilt - Covarrubias - opt

It bothers me when school responsibilities prevent me from helping out at home or participating in family activities.

- Strongly disagree (1)
- Disagree (2)
- Somewhat disagree (3)
- Somewhat agree (4)
- Agree (5)
- Strongly agree (6)

I often avoid talking about school matters and achievements with my family.

○ Strongly disagree (1)

○ Disagree (2)

○ Somewhat disagree (3)

○ Somewhat agree (4)

○ Agree (5)

○ Strongly agree (6)

I feel sad because going to college means many sacrifices by my family.

○ Strongly disagree (1)

○ Disagree (2)

○ Somewhat disagree (3)

○ Somewhat agree (4)

○ Agree (5)

○ Strongly agree (6)

End of Block: Family Achievement Guilt - Covarrubias - opt

Start of Block: Bureaucratic (bureau_frustration)

Think about your experiences with [SCHOOL NAME] so far. In general, how simple or complicated has it been for you to...

	Not at all complicated (1)	Slightly complicated (2)	Somewhat complicate d (3)	Moderately complicated (4)	Quite complicated (5)	Very complicated (6)	Extremely complicat ed (9)	N/A (10)
Figure out which courses you need for your degree or your future career goals?	○	○	○	○	○	○	○	○
Get accurate information about courses or financial aid from the counselors and advisors at [SCHOOL NAME]?	○	○	○	○	○	○	○	○
Apply for financial aid (if applicable)?	○	○	○	○	○	○	○	○
Receive the financial aid and scholarships that you were awarded (if applicable)?	○	○	○	○	○	○	○	○
Register for your courses?	○	○	○	○	○	○	○	○
Complete important paperwork or forms?	○	○	○	○	○	○	○	○

Page Break

Think about your overall experience of trying to get what you need from the offices and administration at [SCHOOL NAME], such as filling out paperwork, getting housing, getting information about your classes or degree plans, or getting financial aid.

In general, how frustrating has your experience been with the offices and administration at [SCHOOL NAME]?

- ○ Not at all frustrating (1)
- ○ Slightly frustrating (2)
- ○ Somewhat frustrating (3)
- ○ Very frustrating (4)
- ○ Extremely frustrating (5)

End of Block: Bureaucratic (bureau_frustration)

Start of Block: Intro: School institution questions

Thank you. The next questions ask about your impressions of DePauw as a whole so far.

End of Block: Intro: School institution questions

Start of Block: System Justification - key

The system at [SCHOOL NAME] is set up so that people usually get the outcome that they deserve.

○ Strongly disagree (1)

○ Disagree (2)

○ Somewhat disagree (3)

○ Neither agree nor disagree (4)

○ Somewhat agree (5)

○ Agree (6)

○ Strongly agree (7)

End of Block: System Justification – key

Start of Block: Representation of Student Groups in Administration (rep_admin)

Please estimate what percentage of the student body at [SCHOOL NAME] is...

- ◯ Women (%) (4) ____
- ◯ Men (%) (5) ____
- ◯ Genderqueer (%) (6) ____
- ◯ Black, African American, or African (%) (7) ____
- ◯ Asian American or Asian (%) (8) ____
- ◯ Latino or Hispanic (%) (9) ____
- ◯ Native American (%) (10) ____
- ◯ Two or more races (%) (11) ____
- ◯ White (%) (12) ____

Page Break

Please estimate what percentage of the faculty at [SCHOOL NAME] is...

- ○ Women (%) (1) ____
- ○ Men (%) (2) ____
- ○ Genderqueer (%) (3) ____
- ○ Black, African American, or African (%) (4) ____
- ○ Asian American or Asian (%) (5) ____
- ○ Latino or Hispanic (%) (6) ____
- ○ Native American (%) (7) ____
- ○ Two or more races (%) (8) ____
- ○ White (%) (9) ____

Page Break

The faculty at [SCHOOL NAME] is ethnically/racially diverse.

○ Strongly agree (1)

○ Agree (2)

○ Mostly agree (3)

○ Mostly disagree (4)

○ Disagree (5)

○ Strongly disagree (6)

Women are well-represented among the faculty on campus.

○ Strongly agree (1)

○ Agree (2)

○ Mostly agree (3)

○ Mostly disagree (4)

○ Disagree (5)

○ Strongly disagree (6)

End of Block: Representation of Student Groups in Administration (rep_admin)

Start of Block: Dialect (dialect)

How different do you speak from how most people speak at [SCHOOL NAME]?

- Not at all different (1)
- A little bit different (2)
- Moderately different (3)
- Quite a bit different (4)
- Extremely different (5)

How much are the ways you prefer to speak and write different from the ways in which it is ideal to speak and write at [SCHOOL NAME]?

- Not at all different (1)
- A little bit different (2)
- Moderately different (3)
- Quite a bit different (4)
- Extremely different (5)

How much are the ways you prefer to speak and write valued by instructors at [SCHOOL NAME]?

- Not valued at all (1)
- A little bit valued (2)
- Moderately valued (3)
- Quite a bit valued (4)
- Extremely valued (5)

How much are the ways you prefer to speak and write valued by your new classmates at [SCHOOL NAME]?

- ○ Not valued at all (1)
- ○ A little bit valued (2)
- ○ Moderately valued (3)
- ○ Quite a bit valued (4)
- ○ Extremely valued (5)

End of Block: Dialect (dialect)

Start of Block: Professors - Fair and Caring (fair_caring)

Professors at [SCHOOL NAME] evaluate me fairly.

- ○ Not true at all (1)
- ○ Slightly true (2)
- ○ Moderately true (3)
- ○ Very true (4)
- ○ Extremely true (5)

Professors at [SCHOOL NAME] care about me as a person.

- Not true at all (1)
- Slightly true (2)
- Moderately true (3)
- Very true (4)
- Extremely true (5)

Professors at [SCHOOL NAME] care about my academic success.

- Not true at all (1)
- Slightly true (2)
- Moderately true (3)
- Very true (4)
- Extremely true (5)

End of Block: Professors - Fair and Caring (fair_caring)

Start of Block: Institutional Growth Mindset - key

Below are some statements about your impression of people at [SCHOOL NAME]. Read each statement and indicate how much you agree or disagree.

In general, most people at [SCHOOL NAME] seem to believe that every student can learn new things and significantly grow their intelligence.

- Strongly agree (1)
- Agree (2)
- Somewhat agree (3)
- Somewhat disagree (4)
- Disagree (5)
- Strongly disagree (6)

So far, it seems that most people at [SCHOOL NAME] believe that students can't really change how intelligent they are.

- Strongly agree (1)
- Agree (2)
- Somewhat agree (3)
- Somewhat disagree (4)
- Disagree (5)
- Strongly disagree (6)

End of Block: Institutional Growth Mindset - key

Start of Block: STEM and non-STEM professors mindsets - key

Now we are going to ask about your perceptions of what professors of different subject areas believe. Again, there are no right or wrong answers, we are just interested in <u>your</u> thoughts about what professors at [SCHOOL NAME] seem to believe.

Page Break

Consider the math and physical science (e.g., physics, chemistry, engineering, computer science, economics) professors at [SCHOOL NAME].

In general, most math and hard science professors at [SCHOOL NAME] seem to believe that some students are smart, while others are not.

- Strongly agree (1)
- Agree (2)
- Somewhat agree (3)
- Somewhat disagree (4)
- Disagree (5)
- Strongly disagree (6)

In general, most math and hard science professors at [SCHOOL NAME] seem to believe that students have a certain amount of intelligence, and they really can't do much to change it.

- Strongly agree (1)
- Agree (2)
- Somewhat agree (3)
- Somewhat disagree (4)
- Disagree (5)
- Strongly disagree (6)

Page Break

Now, please consider the social sciences and humanities (e.g. psychology, history, sociology, english, art) professors at [SCHOOL NAME].

In general, most social science and humanities professors at [SCHOOL NAME] seem to believe that some students are smart, while others are not.

- Strongly agree (1)
- Agree (2)
- Somewhat agree (3)
- Somewhat disagree (4)
- Disagree (5)
- Strongly disagree (6)

In general, most social science and humanities professors at [SCHOOL NAME] seem to believe that students have a certain amount of intelligence, and they really can't do much to change it.

- Strongly agree (1)
- Agree (2)
- Somewhat agree (3)
- Somewhat disagree (4)
- Disagree (5)
- Strongly disagree (6)

End of Block: STEM and non-STEM professors mindsets - key

Start of Block: Intro: health

The next questions are going to ask you about your health and well-being.

End of Block: Intro: health

Start of Block: Health – key

In general, how has your physical health been this past academic year?

○ Excellent (1)

○ Very Good (2)

○ Good (3)

○ Fair (4)

○ Poor (5)

In general, how has your mental health been this past academic year?

○ Excellent (1)

○ Very Good (2)

○ Good (3)

○ Fair (4)

○ Poor (5)

Page Break

End of Block: Health - key

Start of Block: Stress - key

How much stress do you experience on a day-to-day basis at [SCHOOL NAME]?

○ None (1)

○ A little (2)

○ Some (3)

○ A moderate amount (4)

○ A lot (5)

○ A great deal (6)

○ An extreme amount (7)

How confident do you feel that you can handle the stress you experience on a day-to-day basis at [SCHOOL NAME]?

○ Not at all confident (1)

○ Slightly confident (2)

○ Somewhat confident (3)

○ Moderately confident (4)

○ Quite confident (5)

○ Very confident (6)

○ Extremely confident (7)

End of Block: Stress - key

Start of Block: Depression Screener - key

Over the last 2 weeks, how often have you been bothered by the following problems?

	Not at all (1)	Several days (2)	More than half the days (3)	Nearly every day (4)
Feeling nervous, anxious, or on edge (ddepress_1)	○	○	○	○
Not being able to stop or control worrying (ddepress_2)	○	○	○	○
Little interest or pleasure in doing things (ddepress_3)	○	○	○	○
Feeling down, depressed, or hopeless (ddepress_4)	○	○	○	○

End of Block: Depression Screener - key

Start of Block: Height/Weight

Q1019
What is your height in feet and inches? Round to the nearest half of an inch.

▼ 7 feet or above (84) ... 4 feet 10 inches or below (58)

What is your current weight in pounds?

▼ 500 lbs or above (500) ... 90 lbs or below (90)

Page Break

End of Block: Height/Weight

Start of Block: Body Image (body_image)

This academic year I have felt bad about my weight and/or my body shape.

- Strongly disagree (1)
- Disagree (2)
- Somewhat disagree (3)
- Neither agree nor disagree (4)
- Somewhat agree (5)
- Agree (6)
- Strongly agree (7)

This academic year I have tried to lose weight.

- Strongly disagree (9)
- Disagree (10)
- Somewhat disagree (11)
- Neither agree nor disagree (12)
- Somewhat agree (13)
- Agree (14)
- Strongly agree (15)

End of Block: Body Image (body_image)

Start of Block: Commute (commute)

If you are living off campus, how do you commute to campus most of the time?

- Walk (1)
- Bike (2)
- Drive myself (3)
- Get a ride (e.g. carpool, be dropped off) (4)
- Take public transportation (e.g. bus, train) (5)
- Other (6) ____

Page Break

Display This Question:

If __pdd__t2__demog__commute__pdd__ If you are living off campus, how do you commute to campus most... = Walk

Or __pdd__t2__demog__commute__pdd__ If you are living off campus, how do you commute to campus most... = Bike

Or __pdd__t2__demog__commute__pdd__ If you are living off campus, how do you commute to campus most... = Drive myself

Or __pdd__t2__demog__commute__pdd__ If you are living off campus, how do you commute to campus most... = Get a ride (e.g. carpool, be dropped off)

Or __pdd__t2__demog__commute__pdd__ If you are living off campus, how do you commute to campus most... = Take public transportation (e.g. bus, train)

Or __pdd__t2__demog__commute__pdd__ If you are living off campus, how do you commute to campus most... = Other

How many minutes does your commute to [SCHOOL NAME] take on most days?

____

End of Block: Commute (commute)

Start of Block: Job (job)

Are you working in a paid position?

○ Yes, a full-time job (30 hours a week or more) (1)

○ Yes, a part-time job (less than 30 hours a week) (2)

○ No (0)

End of Block: Job (job)

Start of Block: Intro: Identity Section

Thank you. The next questions ask more about yourself, different types of identities people have (e.g. ethnic, gender, urban/rural, other), and experiences people might have based on these identities.

End of Block: Intro: Identity Section

Start of Block: Religion (religion)

Over the past month of school, how close have you felt to God, gods, or a higher power?

- ○ N/A; I do not believe in a higher power (0)
- ○ Not at all close (1)
- ○ Somewhat close (2)
- ○ Close (3)
- ○ Very close (4)
- ○ Extremely close (5)

In the past month of school, how often have you had struggles related to religion or spirituality?

Struggles can include: feelings of confusion or doubt about your religious/spiritual beliefs; feeling as though your life had no deeper meaning; conflicts with other people about religious/spiritual matters or feeling hurt/mistreated by religious people; or feeling attacked by evil force.

- ○ Never/not at all (1)
- ○ Rarely (2)
- ○ Occasionally (3)
- ○ Often (4)
- ○ Very Often (5)

Page Break

With what religious group do you most closely identify?

○ Agnostic (not sure if there is a God) (13)

○ Atheist (do not believe in God) (14)

○ Baha'i (15)

○ Buddhist (1)

○ Christian - Catholic (2)

○ Christian - Orthodox (e.g., Eastern Orthodox, Greek Orthodox, Russian Orthodox, or some other Orthodox church) (3)

○ Christian - Jehovah's Witness (16)

○ Christian - Mormon (Church of Jesus Christ of Latter-day Saints/LDS) (4)

○ Christian- Protestant (e.g., Methodist, Pentecostal, Baptist, Presbyterian, Non-denominational, Anglican, Lutheran) (5)

○ Hindu (6)

○ Jewish (7)

○ Muslim (8)

○ Native American or indigenous religion (9)

○ Sikh (10)

○ Taoist (17)

○ Unitarian (Universalist) (18)

○ Wiccan (19)

○ Spiritual but not religious (11)

○ Not spiritual or religious (20)

○ Other (12) ____

End of Block: Religion (religion)

Start of Block: Urban/Rural (rural)

Do you think of yourself as having grown up in an urban, suburban, or rural place?

○ Urban (1)

○ Suburban (2)

○ Rural (3)

○ Other (4) ____

End of Block: Urban/Rural (rural)

Start of Block: Athlete (athlete)

Are you currently a student athlete?

○ Yes (1)

○ No (0)

End of Block: Athlete (athlete)

Start of Block: Athlete - extras (athlete)

What is your sport?

____

Page Break

Please indicate how true each of the following statements are for you:

My identity as a student is an important part of who I am.

- Not at all true (1)
- A little bit true (2)
- Somewhat true (3)
- Pretty true (4)
- Very true (5)
- Extremely true (6)

My identity as an athlete is an important part of who I am.

- Not at all true (1)
- A little bit true (2)
- Somewhat true (3)
- Pretty true (4)
- Very true (5)
- Extremely true (6)

Page Break

I worry that I will be negatively judged in college because I'm an athlete.

- Not at all true (1)
- A little bit true (2)
- Somewhat true (3)
- Pretty true (4)
- Very true (5)
- Extremely true (6)

I believe that my experiences and skills as an athlete will help me succeed as a student.

○ Not at all true (1)

○ A little bit true (2)

○ Somewhat true (3)

○ Pretty true (4)

○ Very true (5)

○ Extremely true (6)

Page Break

You have a certain amount of athletic ability, and you can't really do much to change it.

○ Strongly agree (1)

○ Agree (2)

○ Mostly agree (3)

○ Mostly disagree (4)

○ Disagree (5)

○ Strongly disagree (6)

You can grow your basic athletic ability a lot in your lifetime.

○ Strongly agree (1)

○ Agree (2)

○ Mostly agree (3)

○ Mostly disagree (4)

○ Disagree (5)

○ Strongly disagree (6)

End of Block: Athlete - extras (athlete)

Start of Block: Gender/Racial Identification - key

Please indicate the extent to which you agree or disagree with each statement using the scales below. Please use the whole range of each scale.

My gender identity is an important part of who I am.

- ○ Not at all true (1)
- ○ Slightly true (2)
- ○ Moderately true (3)
- ○ Very true (4)
- ○ Extremely true (5)

My racial/ethnic identity is an important part of who I am.

- ○ Not at all true (1)
- ○ Slightly true (2)
- ○ Moderately true (3)
- ○ Very true (4)
- ○ Extremely true (5)

End of Block: Gender/Racial Identification - key

Start of Block: Stereotype Threat - key

Do you think other people at your school would be surprised or not surprised if you or people like you succeeded in school?

- ○ Extremely surprised (1)
- ○ Very surprised (2)
- ○ Moderately surprised (3)
- ○ Slightly surprised (4)
- ○ Not surprised (5)

Page Break

At [SCHOOL NAME], to what extent do you worry that people negatively judge you based on what they think about your racial group?

- Not at all (1)
- A little bit (2)
- Somewhat (3)
- A moderate amount (4)
- A lot (5)
- A great deal (6)
- An extreme amount (7)

At [SCHOOL NAME], to what extent do you worry that people negatively judge you based on what they think about your social class background?

- Not at all (1)
- A little bit (2)
- Somewhat (3)
- A moderate amount (4)
- A lot (5)
- A great deal (6)
- An extreme amount (7)

In math and science classes at [SCHOOL NAME], to what extent do you worry that people negatively judge you based on what they think about your gender group?

- Not at all (1)
- A little bit (2)
- Somewhat (3)
- A moderate amount (4)
- A lot (5)
- A great deal (6)
- An extreme amount (7)

End of Block: Stereotype Threat - key

Start of Block: [SCHOOL NAME] Stereotype Threat Questions

In history and social science classes at [SCHOOL NAME], to what extent do you worry that people negatively judge you, based on what they think about your gender group?

- Not at all (1)
- A little (2)
- Somewhat (3)
- A moderate amount (4)
- A lot (5)
- A great deal (6)
- An extreme amount (7)

Page Break

In arts and humanities classes at [SCHOOL NAME], to what extent do you worry that people negatively judge you, based on what they think about your gender group?

- Not at all (1)
- A little (2)
- Somewhat (3)
- A moderate amount (4)
- A lot (5)
- A great deal (6)
- An extreme amount (7)

End of Block: [SCHOOL NAME] Stereotype Threat Questions

Start of Block: Contextual threat - key

To what extent do minority-group students (e.g. racial, ethnic, sexual, religious minorities) experience bias, discrimination, or unfair treatment at [SCHOOL NAME]?

- Not at all (1)
- A little bit (2)
- Somewhat (3)
- A moderate amount (4)
- A lot (5)
- A great deal (6)
- An extreme amount (7)

End of Block: Contextual threat - key

Start of Block: Brenda Majors Stereotype Threat

Since arriving at [SCHOOL NAME], how often have you been treated unfairly because of your race or ethnicity?

- ◯ Never (1)
- ◯ Less than once a year (2)
- ◯ A few times a year (3)
- ◯ A few times a month (4)
- ◯ At least once a week (5)
- ◯ Almost every day (6)

Since arriving at [SCHOOL NAME], how often have you been treated unfairly because of your social class background?

- ◯ Never (1)
- ◯ Less than once a year (2)
- ◯ A few times a year (3)
- ◯ A few times a month (4)
- ◯ At least once a week (5)
- ◯ Almost every day (6)

End of Block: Brenda Majors Stereotype Threat

Start of Block: Race/Collective Action (collective_action)

I believe that most people at [SCHOOL NAME] genuinely want to make [SCHOOL NAME] a more inclusive place with regards to race and class.

- Strongly disagree (1)
- Disagree (2)
- Somewhat disagree (3)
- Neither agree nor disagree (4)
- Somewhat agree (5)
- Agree (6)
- Strongly agree (7)

Inequalities due to race and class are deeply entrenched at [SCHOOL NAME].

- Strongly disagree (1)
- Disagree (2)
- Somewhat disagree (3)
- Neither agree nor disagree (4)
- Somewhat agree (5)
- Agree (6)
- Strongly agree (7)

I believe inequalities due to race and class at [SCHOOL NAME] will improve during my time here.

- ○ I do not believe there are inequalities due to race and class at [SCHOOL NAME] (1)
- ○ Strongly disagree (2)
- ○ Disagree (3)
- ○ Somewhat disagree (4)
- ○ Neither agree nor disagree (5)
- ○ Somewhat agree (6)
- ○ Agree (7)
- ○ Strongly agree (11)

Page Break

Overall, how engaged have you been this year in activism or efforts to make [SCHOOL NAME] a more inclusive place?

- Not at all engaged (1)
- A little engaged (2)
- Somewhat engaged (3)
- Moderately engage (4)
- Quite engaged (5)
- Very engaged (6)
- Extremely engaged (7)

Page Break

Overall, how engaged are you in organizations or groups to help, empower, or serve other people (e.g., community service, social justice, volunteering)?

- Not at all engaged (1)
- A little engaged (2)
- Somewhat engaged (3)
- Moderately engaged (4)
- Quite engaged (5)
- Very engaged (6)
- Extremely engaged (7)

End of Block: Race/Collective Action (collective_action)

Start of Block: Intro: Happiness/Overall Questions

Thank you. The last questions ask about your life overall at [SCHOOL NAME].

End of Block: Intro: Happiness/Overall Questions

Start of Block: Short Flourishing - key

Right now, how much do you feel that your life at [SCHOOL NAME] has a sense of direction or meaning to it?

- ○ Not at all (1)
- ○ A little (2)
- ○ A moderate amount (3)
- ○ A lot (4)
- ○ A great deal (5)

Page Break

End of Block: Short Flourishing - key

Start of Block: Overall Positivity of School Year – key

Looking back, all things considered, on the whole how positive or negative has your first year at [SCHOOL NAME] been?

- Extremely negative (1)
- Very negative (2)
- Quite negative (3)
- Somewhat negative (4)
- Slightly negative (5)
- Slightly positive (6)
- Somewhat positive (7)
- Quite positive (8)
- Very positive (9)
- Extremely positive (10)

End of Block: Overall Positivity of School Year - key

Start of Block: Happiness/Wellbeing/Life Satisfaction – key

All things considered, how satisfied are you with your life as a whole?

- Totally dissatisfied 1 (1)
- 2 (2)
- 3 (3)
- 4 (4)
- 5 (5)
- 6 (6)
- 7 (7)
- 8 (8)
- 9 (9)
- Totally satisfied 10 (10)

Page Break

In general, I consider myself:

◯ Not a very happy person 1 (1)

◯ 2 (2)

◯ 3 (3)

◯ 4 (4)

◯ 5 (5)

◯ 6 (6)

◯ A very happy person 7 (7)

Compared with most of my peers, I consider myself:

◯ Less happy 1 (1)

◯ 2 (2)

◯ 3 (3)

◯ 4 (4)

◯ 5 (5)

◯ 6 (6)

◯ More happy 7 (7)

End of Block: Happiness/Wellbeing/Life Satisfaction - key

Start of Block: Retention - key

Do you plan to attend [SCHOOL NAME] next fall?

◯ Yes (1)

◯ Unsure/undecided (2)

◯ No (3)

Page Break

Display This Question:

If __pdd__t2__dv__attend_fall__pdd__ Do you plan to attend [SCHOOL NAME] next fall? = Unsure/undecided

Or __pdd__t2__dv__attend_fall__pdd__ Do you plan to attend [SCHOOL NAME] next fall? = No

Please select all of the reasons below that have led you to consider stopping your education here:

- ☐ I am transferring to another school (1)
- ☐ Financial problems (2)
- ☐ Academic difficulties (3)
- ☐ I don't feel like I fit in here (4)
- ☐ The bureaucracy here is too difficult to navigate (5)
- ☐ I miss my home/family (6)
- ☐ Physical/mental health problems (7)
- ☐ I just don't like being in school (8)
- ☐ Other (9) ____
- ☐ I will be working on a political campaign. (10)
- ☐ I'm going to take some time off to work on a start-up or new business. (11)
- ☐ Other (12) ____

End of Block: Retention – key

Start of Block: [SCHOOL NAME] retention addition

Display This Question:

If Please select all of the reasons below that have led you to consider stopping your education here: = I am transferring to another school

Where are you transferring to?

- A 2-year school in [STATE] (1)
- A 4-year school in [STATE] (2)
- A 2-year school outside of [STATE] (3)
- A 4-year school outside of [STATE] (4)

End of Block: [SCHOOL NAME] retention addition

Start of Block: Open Ended - key

We would like to learn more about what [SCHOOL NAME] has been like for you this year. Please spend the next few minutes writing about your experience this year.

There's no need to write at length but please write enough so that we have an overall sense of what your experience has been like. Don't worry about spelling or punctuation.

Thank you for taking your time. Learning more about your experience will help us understand more about what it is like for students to come to [SCHOOL NAME] and how we can improve this transition for future students. (The "next" button will appear after 90 seconds. You may continue writing as long as you like. When you are done, click "next.")

____

____

____

____

____

End of Block: Open Ended - key

Start of Block: Open Ended - advice (open_advice)

What advice would you give an incoming student next year to help them have a successful/positive experience at [SCHOOL NAME]?

____

____

____

____

____

End of Block: Open Ended - advice (open_advice)

Start of Block: Fidelity Check - key

Please answer two final questions about your experience completing this survey.

How distracted were you as you answered the questions? (e.g., by interruptions, other people, social media, etc.)

○ Not at all distracted (1)

○ Slightly distracted (2)

○ Somewhat distracted (3)

○ Very distracted (4)

○ Extremely distracted (5)

Did you have any technical difficulties as you completed the survey? For instance, did you have to restart the questions, did your computer freeze up, did the internet stop working, or did anything else happen that interfered with your ability to complete it?

○ Yes, I had some technical difficulties with the survey. *Please explain what happened.* (1) ____

○ No, everything worked fine. (0)

End of Block: Fidelity Check - key

Start of Block: Email Address and Thank-you Note

Thank you for completing this survey!

Your responses will help us make the transition to college better for future generations of [SCHOOL NAME] students.

End of Block: Email Address and Thank-you Note
